# Corticotropin-Releasing Factor Receptor-1 Neurons in the Lateral Amygdala Display Selective Sensitivity to Acute and Chronic Ethanol Exposure

**DOI:** 10.1523/ENEURO.0420-19.2020

**Published:** 2020-03-02

**Authors:** Abigail E. Agoglia, ManHua Zhu, Rose Ying, Harpreet Sidhu, Luis A. Natividad, Sarah A. Wolfe, Matthew W. Buczynski, Candice Contet, Loren H. Parsons, Marisa Roberto, Melissa A. Herman

**Affiliations:** 1 Department of Pharmacology, School of Medicine, University of North Carolina at Chapel Hill, Chapel Hill, North Carolina 27599; 2 Bowles Center for Alcohol Studies, School of Medicine, University of North Carolina at Chapel Hill, Chapel Hill, North Carolina 27599; 3 Neuroscience Curriculum, School of Medicine, University of North Carolina at Chapel Hill, Chapel Hill, North Carolina 27599; 4 Department of Neuroscience, The Scripps Research Institute, La Jolla, California 92037; 5 University of Texas at Austin, College of Pharmacy - Division of Pharmacology and Toxicology, Austin, TX 78712; 6 School of Neuroscience, Virginia Polytechnic Institute and State University, Blacksburg, Virginia 24061

**Keywords:** alcohol, basolateral amygdala, CRF, CRF1 receptor, GABA, lateral amygdala

## Abstract

The lateral amygdala (LA) serves as the point of entry for sensory information within the amygdala complex, a structure that plays a critical role in emotional processes and has been implicated in alcohol use disorders. Within the amygdala, the corticotropin-releasing factor (CRF) system has been shown to mediate some of the effects of both stress and ethanol, but the effects of ethanol on specific CRF1 receptor circuits in the amygdala have not been fully established. We used male CRF1:GFP reporter mice to characterize CRF1-expressing (CRF1^+^) and nonexpressing (CRF1^−^) LA neurons and investigate the effects of acute and chronic ethanol exposure on these populations. The CRF1^+^ population was found to be composed predominantly of glutamatergic projection neurons with a minority subpopulation of interneurons. CRF1^+^ neurons exhibited a tonic conductance that was insensitive to acute ethanol. CRF1^−^ neurons did not display a basal tonic conductance, but the application of acute ethanol induced a δ GABA_A_ receptor subunit-dependent tonic conductance and enhanced phasic GABA release onto these cells. Chronic ethanol increased CRF1^+^ neuronal excitability but did not significantly alter phasic or tonic GABA signaling in either CRF1^+^ or CRF1^−^ cells. Chronic ethanol and withdrawal also did not alter basal extracellular GABA or glutamate transmitter levels in the LA/BLA and did not alter the sensitivity of GABA or glutamate to acute ethanol-induced increases in transmitter release. Together, these results provide the first characterization of the CRF1^+^ population of LA neurons and suggest mechanisms for differential acute ethanol sensitivity within this region.

## Significance Statement

The corticotropin-releasing factor (CRF) system is a critical component of the stress network and has been implicated in psychiatric disorders including addiction, anxiety, and depression. The present study examines CRF receptor-1 (CRF1) lateral amygdala (LA) neurons and reports differential inhibitory control and acute ethanol effects of CRF1 LA neurons compared with the unlabeled (CRF1^−^) population. An improved understanding of CRF1 amygdala circuitry and the selective sensitivity of that circuitry to ethanol represents an important step in identifying brain region-specific neuroadaptations that occur with ethanol exposure. The present findings also have broad implications, including potential relevance to the role of CRF1 circuitry in other contexts that may provide insight into other disorders involving amygdala dysfunction, including anxiety and depression.

## Introduction

The amygdala complex has been implicated in a number of important functions, notably emotional processing of internal and external sensory stimuli and the coordination of relevant behavioral output (Pitkänen et al., 1997). Amygdala dysfunction is implicated in anxiety (Tye et al., 2011) and alcohol abuse disorders (Koob et al., 1998). The lateral amygdala (LA) serves as the entry point for sensory information and sends excitatory projections to other amygdala nuclei, including the central amygdala (CeA) and basolateral amygdala (BLA), to facilitate stimuli processing (Sah et al., 2003; Agoglia and Herman, 2018). The LA is required for the acquisition and expression of fear learning and memory (Sears et al., 2014), and plays a crucial role in the development of anxiety-like behaviors (Rodrigues et al., 2004). Similar mechanisms may be involved in the dysregulated amygdalar activity seen in alcohol dependence (McCool et al., 2010), but the diversity of cell types within the LA complicates the interpretation of ethanol (EtOH) effects.

GABAergic neurotransmission is sensitive to acute and chronic ethanol exposure, and GABA_A_ receptor activity is involved in ethanol tolerance and dependence. (Eckardt et al., 1998; Grobin et al., 1998; Weiner and Valenzuela, 2006). Both phasic (immediate, short-term inhibition) and tonic (persistent inhibition) GABAergic transmission within the CeA is sensitive to acute and chronic ethanol in a cell type-specific manner (Herman et al., 2013, 2016). The functional characteristics of GABA_A_ receptors are determined by their subunit composition; receptors containing the α4, α6, and/or δ subunit are expressed extrasynaptically and mediate tonic conductance (Semyanov et al., 2004). These receptors also display an increased sensitivity to ethanol (Wallner et al., 2003; Wei et al., 2004) and may be a primary target for ethanol in the brain (Wallner et al., 2003; Mody et al., 2007), although the direct action of ethanol on tonic GABA_A_ receptors remains controversial (Borghese and Harris, 2007; Baur et al., 2009). Tonic inhibition has been described in principal cells and local interneurons in the LA, but the receptor composition mediating this tonic conductance in LA neurons is unclear (Marowsky et al., 2012).

Corticotropin-releasing factor (CRF) and the CRF receptor-1 (CRF1) are expressed throughout the amygdala (Van Pett et al., 2000; Calakos et al., 2017) and have been implicated in neuroplastic changes related to fear (Hubbard et al., 2007), anxiety (Overstreet et al., 2004; Rainnie et al., 2004), and alcohol exposure (Nie et al., 2004; Roberto et al., 2010; Herman et al., 2013; Lovinger and Roberto, 2013). Notably, activation of CRF1 receptors increases the excitability of BLA neurons to sensory input (Ugolini et al., 2008), and administration of CRF into the BLA increases activation of calcium/calmodulin-dependent protein kinase II (CaMKII)-containing projection neurons (Rostkowski et al., 2013). Despite the expression of CRF and CRF1 in the LA and the relevance of the CRF system to the consequences of ethanol exposure, the specific effects of ethanol on the LA CRF1 neuronal population have not been characterized.

Previous work using a transgenic mouse line expressing green fluorescent protein (GFP) under the *Crhr1* promoter (Justice et al., 2008) found that CRF1^+^ and CRF1^−^ neurons within the CeA exhibit distinct inhibitory characteristics and differential sensitivity to acute and chronic ethanol (Herman et al., 2013, 2016). The CRF1-containing neuronal population within the LA has not been previously characterized, and could be an important determinant of LA activity and output as well as a site of action for drugs of abuse such as ethanol. The current study uses the same CRF1:GFP mice to selectively target and characterize CRF1 neurons in the LA, not to probe the effect of CRF1 activation, which will be the subject of future studies. Here, we combine electrophysiology, immunohistochemistry, and microdialysis to (1) characterize the phenotype of CRF1^+^ and CRF1^−^ neurons of the LA, (2) investigate phasic and tonic inhibitory transmission in LA CRF1^+^ and CRF1^−^ cells, and (3) determine the effects of acute and chronic ethanol exposure on inhibitory control within the LA.

## Materials and Methods

### Animals

Experiments were performed in 59 adult (age, 3–6 months; weight, 19–30 g) male transgenic CRF1:GFP mice that express GFP under the *Crhr1* promoter, as previously described (Justice et al., 2008). Mice were bred and group housed in a temperature- and humidity-controlled 12 h light/dark facility with *ad libitum* access to food and water. All experiments were performed in tissue collected from mice between zeitgeber 2 and 7. All procedures were approved by the Scripps Research Institute and the University of North Carolina at Chapel Hill Institutional Animal Care and Use Committees.

### Electrophysiological recording

Coronal sections (300 μm) were prepared with a Leica VT1000S (Leica Microsystems) from brains that were rapidly extracted from mice after brief anesthesia (5% isoflurane) and placed in ice-cold sucrose solution containing (in mm): sucrose 206.0; KCl 2.5; CaCl_2_ 0.5; MgCl_2_ 7.0; NaH_2_PO_4_ 1.2; NaHCO_3_ 26; glucose 5.0; and HEPES 5. After sectioning, slices were incubated in an oxygenated (95% O_2_/5% CO_2_) artificial CSF (aCSF) solution containing (in mm): NaCl 130, KCl 3.5, NaH2PO4 1.25, MgSO4 1.5, CaCl2 2, NaHCO3 24, and glucose 10 for 30 min at 37°C, followed by 30 min equilibration at room temperature (RT; 21–22°C). Recordings were made with patch pipettes (3–6 MΏ; Warner Instruments) filled with an intracellular solution containing (in mm): KCl 145; EGTA 5; MgCl_2_ 5; HEPES 10; Na-ATP 2; and Na-GTP 0.2, coupled to a Multiclamp 700B amplifier (Molecular Devices), acquired at 10 kHz, low-pass filtered at 2–5 kHz, digitized at 20 kHz (Digidata 1440A digitizer; Molecular Devices), and stored on a computer using pClamp 10 software (Axon Instruments). Series resistance was typically <15 MΩ and was continuously monitored with a hyperpolarizing 10 mV pulse; neurons with series resistance >15 MΩ or >20% change in resistance during recording were excluded from final analysis. LA neurons containing the CRF1 receptor were identified by GFP expression and differentiated from unlabeled (GFP^−^) neurons using fluorescent optics and brief (<2 s) episcopic illumination in slices from CRF1:GFP reporter mice. Electrophysiological properties of cells were determined by pClamp 10 Clampex software online during voltage-clamp recording using a 10 mV pulse delivered after breaking into the cell. Drugs were applied either by bath or Y-tube application for local perfusion. Recordings (V_hold_ = −60 mV) were performed in the presence of the glutamate receptor blockers 6,7-dinitroquinoxaline-2,3-dione (DNQX; 20 μm) and aminophosphonopentanoic acid (AP-5; 50 μm) and the GABA_B_ receptor antagonist CGP55845A (1 μm). All recordings were conducted at room temperature, and all solutions (bath and Y-tube) were prepared and maintained at room temperature.

### Drugs and chemicals

DNQX (10 μm), AP-5 (50 μm), and CGP55845A (1 μm) were purchased from Tocris Bioscience. SR-95 531 [gabazine (GBZ); 100 μm], picrotoxin (100 μm), and 4,5,6,7-tetrahydroisoxazolo[5,4-c]pyridin-3-ol (THIP; 1–10 μm) were purchased from Sigma-Aldrich.

### Immunohistochemistry

Mice (*n* = 4) were anesthetized with isoflurane and perfused with cold PBS followed by 4% paraformaldehyde (PFA). Brains were dissected and immersion fixed in PFA for 24 h at 4°C, cryoprotected in sterile 30% sucrose in PBS for 24–48 h at 4°C or until brains sank, flash frozen in prechilled isopentane on dry ice, and stored at −80°C. Free-floating 35 μm brain sections were obtained using a cryostat and kept at 4°C in PBS containing 0.01% sodium azide.

Sections were washed in PBS for 10 min at RT with gentle agitation and then blocked for 1 h at RT in blocking solution (0.3% Triton X-100, 1 mg/ml bovine serum albumin, and 5% normal goat serum (NGS)]. Primary antibody was incubated at 4°C overnight with gentle agitation in 0.5% Tween-20 and 5% NGS. The following primary antibodies were used: chicken anti-GFP (1:2000; catalog #ab13970, Abcam; RRID:AB_300798); rabbit anti-α1 and rabbit anti-δ GABA_A_ receptor subunit (1:100; 812-GA1N, 868A-GDN, PhosphoSolutions); mouse anti-parvalbumin (PV; 1:1000; catalog #235, Swant; RRID:AB_10000343); mouse anti-calretinin (1:500; catalog #6B3, Swant; RRID:AB_10000320); and mouse anti-calbindin (1:2000; catalog #300, Swant, RRID:AB_10000347). Antibodies against native mouse protein were validated by the manufacturer with tissue from knock-out mice, with the exception of anti-δ GABA_A_. Next, sections were triple washed in PBS for 10 min at RT with gentle agitation followed by a 1 h secondary antibody incubation in PBS (in the dark). The following secondary antibodies were used: Alexa Fluor 488 goat anti-chicken (catalog #A-11039, Thermo Fisher Scientific; RRID:AB_142924); Cy-3 donkey anti-rabbit (catalog #711–165-152, Jackson ImmunoResearch; RRID:AB_2307443); and Alexa Fluor 568 goat anti-mouse (catalog #A-11004, Thermo Fisher Scientific; RRID:AB_2534072). Sections were then washed (10 min, RT, three times) and mounted in Vectashield (catalog #H1500, Vector Laboratories; RRID:AB_2336788).

Sections were imaged on a Zeiss LSM 780 laser-scanning confocal microscope (10× objective, tile scanning of LA). All microscope settings were kept the same within experiments during image acquisition. The analyst was blind to the identity of the red fluorescent signal when performing cell counts, and analysis was performed manually in an unbiased manner at four anterior–posterior levels (equidistant sections located −1.00 through −1.70 mm from bregma). Data are presented as the mean ± SE.

### *In situ* hybridization

Mice (*n* = 3) were perfused with ice cold PBS/Z-fix (catalog #NC9378601, Thermo Fisher Scientific) after anesthesia with isoflurane. Following perfusion, brains were dissected and immersion fixed for 24 h in Z-fix at 4°C, cryoprotected in 30% sucrose in PBS for 24 h at 4°C, and flash frozen in isopentane on dry ice. Brains were preliminarily stored at −80°C until they were sliced on a cryostat in 20-μm-thick sections, mounted on SuperFrost Plus slides (catalog #1255015, Thermo Fisher Scientific), and stored at −80°C.

Using an RNAscope fluorescent multiplex kit (catalog #320850, ACD), *in situ* hybridization was performed for *Crhr1*, *Gfp*, and *Slc17a7*. Target retrieval pretreatment as outlined in the manual provided by RNAscope (document #320535, ACD) was performed by first briefly washing prepared slides in PBS. Next slides were submerged in prewarmed target retrieval buffer (catalog #322000, ACD) and kept at a constant temperature between 95°C and 98°C for 10 min. Slides were then removed and immediately rinsed in distilled water twice, and then dehydrated with 100% ethanol. After dehydrations, slices were demarcated with a hydrophobic barrier pen (catalog #310018, ACD) and digested with Protease IV for 20 min at 40°C in a hybridization oven. Next, the RNAscope Fluorescent Multiplex Reagent Kit User Manual (document #320293, ACD) was followed entirely. Last, slides were mounted with Vectashield with DAPI (catalog #NC9029229, Thermo Fisher Scientific). The probes used were *Crhr1* (probe target region 207–813; catalog #418011-C2), *Slc17a7* (probe target region 464–1415; catalog #416631-C1), eGFP (probe target region 628–1352; catalog #400281), and a negative control (catalog #320751), all from ACD.

Slides were imaged on a Zeiss LSM 780 laser-scanning confocal microscope (40× oil-immersion, 1024 × 10^24^; of LA at approximately −1.46 mm from bregma; 5 μm *z*-stacks). All microscope settings were kept the same within experiments during image acquisition. Background was subtracted from images based on the negative control for each probe, and signal intensity present in DAPI-labeled nuclei after background subtracted denoted positive cells. To perform quantification, ImageJ was used to manually count DAPI-labeled nuclei expressing fluorescently labeled probes in the region of interest (ROI). Next, the percentage of nuclei positive for one or both probes and the percentage of signal colocalization were calculated. The percentage of *Crhr1*^+^ nuclei expressing a marker of interest was determined by dividing the number of colabeled nuclei by the total number of *Crhr1*^+^ nuclei. Quantification was performed on three to four images (approximately −1.46 mm from bregma) from each mouse in an unbiased manner as probe fluorescence was quantified blindly. Brightness/contrast and pixel dilation are the same for all representative images.

### Chronic intermittent ethanol vapor inhalation

Mice were placed in ethanol inhalation chambers (La Jolla Alcohol Research) and exposed to chronic intermittent ethanol (CIE) vapor (16 h) followed by air (8 h) daily for 4 consecutive days/week for a period of 4–5 weeks (Herman et al., 2016). Before each vapor exposure, CIE mice were injected with a solution of ethanol (1.5 g/kg) and pyrazole (1 mmol/kg, i.p.), an alcohol dehydrogenase inhibitor, to initiate intoxication and maintain constant blood alcohol levels (BALs). Control mice were exposed to room air and received an injection of pyrazole (1 mmol/kg, i.p.) at the onset of each ethanol vapor exposure. Ethanol drip rate and air flow were adjusted so as to yield BALs averaging 100–250 mg/dl. BALs were measured throughout exposure using an Analox GM7 analyzer. Average BALs for the CIE mice included in electrophysiological recordings were 174.6 ± 15.5 mg/dl. Average BALs for CIE mice in microdialysis experiments were 162.7 ±16.5 mg/dl. Terminal BALs were also determined at the time of death when mice were killed immediately after their last ethanol vapor exposure (CIE mice). Another group of mice underwent 3–7 d of withdrawal after their last vapor exposure before being killed (CIE-WD mice).

### Microdialysis

Mice (*n* = 11) were unilaterally implanted with custom fabricated microdialysis probes (0.5 mm regenerated cellulose) aimed at the LA (from bregma: anteroposterior, −1.5 mm; mediolateral, ±2.9 mm; dorsoventral, −4.1 mm from dura). However, as some penetrance into BLA is possible, microdialysis results are described throughout as LA/BLA. Mice were perfused with aCSF at 0.2 μl/min and allowed to recover overnight, as previously described (Pavon et al., 2018, 2019). The following morning, the flow rate was increased to 0.6 μl/min and allowed to equilibrate for 60 min prior to collection. Dialysate samples were collected at 15 min intervals during a 1.5 h baseline period. Ethanol (1 m) was added to the aCSF perfusate solution for reverse dialysis through the probe, and samples were collected for an additional 1.5 h during the local ethanol exposure period. This dose of ethanol was chosen for consistency with prior experiments using reverse dialysis in rodents, where 1 m was found to induce maximal changes in extracellular GABA and glutamate levels (Roberto et al., 2004a,b).

Quantification of neurotransmitters was performed using triple liquid chromatography quadrupole mass spectrometry methods as previously described (Song et al., 2012; Buczynski et al., 2016). Briefly, microdialysate samples (5 μl) were derivatized with 100 mm borate (5 μl), 2% benzoyl chloride (2 μl, in acetonitrile), and 1% formic acid (2 μl), and were subsequently spiked with benzoylated ^13^C_6_-labeled internal standards (5 μl, in 98% v/v ACN, 1% formic acid, and 1% H_2_O). Samples (10 μl, 4°C) were separated by high-performance liquid chromatography and analyzed by positive-ion mode tandem quadrupole mass spectrometry (catalog #6460 QQQ, Agilent) using multiple-reaction monitoring. The following neurotransmitters were quantified using the standard isotope dilution method (precursor → product): the amino acids aspartate (238 → 105), GABA (208 → 105), glutamate (252 → 105), glutamine (251 → 105), glycine (180 → 105), serine (210 → 105), and taurine (230 → 105). Baseline concentrations were expressed as an absolute value (nanomolar), while changes produced by ethanol reverse dialysis were expressed as relative values (percentage of baseline) over time.

### Statistical analysis

Membrane characteristics and excitability were compared between groups using a two-tailed *t* test or a one-way ANOVA, where appropriate. Frequency, amplitude, and decay of spontaneous IPSCs (sIPSCs) were analyzed and visually confirmed using a semiautomated threshold-based mini detection software (Mini Analysis, Synaptosoft). sIPSC characteristics were determined from baseline and experimental drug conditions containing a minimum of 60 events (time period of analysis varied as a product of individual event frequency). All detected events were used for analysis, and superimposed events were eliminated. Tonic conductance was determined using Clampfit 10.2 (Molecular Devices) and a previously described method (Belelli et al., 2009) in which the mean holding current (i.e., the current required to maintain the −60 mV membrane potential) was obtained by a Gaussian fit to an all-points histogram over a 5 s interval. The all-points histogram was constrained to eliminate the contribution of sIPSCs to the holding current. Drug responses were quantified as the difference in holding current between baseline and experimental conditions. Events were analyzed for independent significance using a one-sample *t* test and compared using a two-tailed *t* test for independent samples, a paired two-tailed *t* test for comparisons made within the same recording, and a one-way ANOVA for comparisons made among three or more groups. In the microdialysis experiments, average baseline concentrations of glutamate and GABA were compared in CIE-WD versus AIR controls using two-tailed *t* tests. To examine the effects of acute ethanol administration on LA/BLA dialysate, two-way repeated-measures ANOVA (exposure condition × time) was used to compare air to CIE-WD mice before and after reverse dialysis of ethanol. All statistical analyses were performed using Prism version 5.02 (GraphPad Software). Data are presented as mean ± SE. In all cases, *p* < 0.05 was the criterion for statistical significance.

## Results

### Phenotype of CRF1^+^ LA neurons

**Figure 1.**
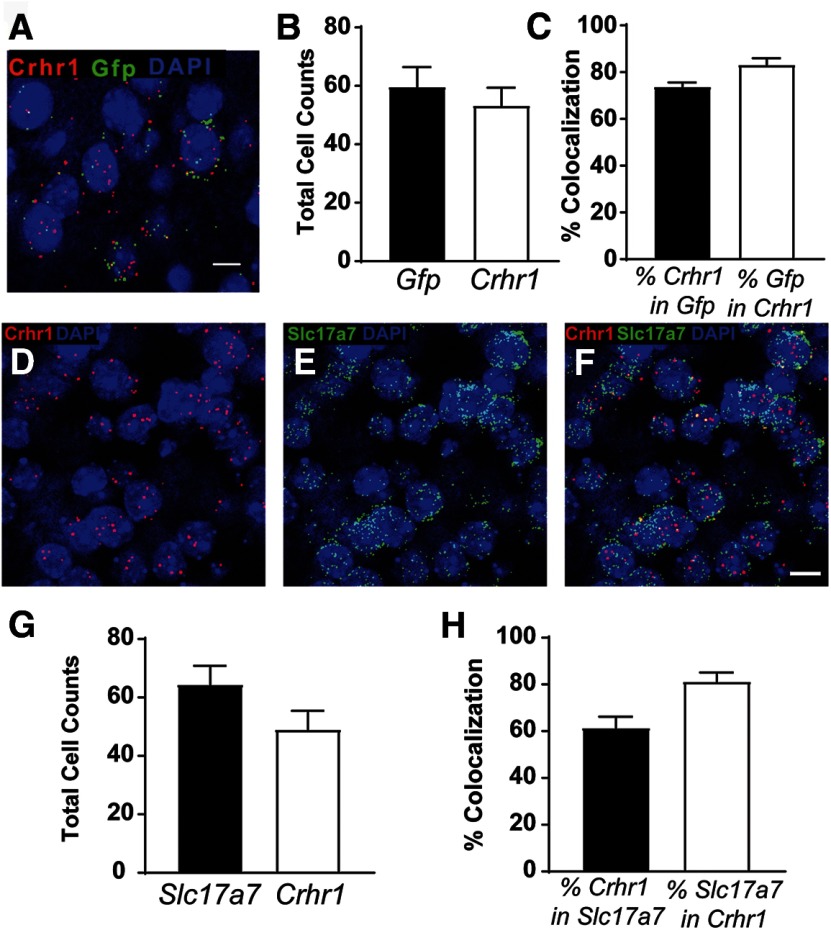
Glutamate transporter expression in CRF1 lateral amygdala neurons. ***A***, Representative merged image showing *Crhr1*, *Gfp*, and DAPI in the LA. Scale bar, 10 μm. ***B***, Summary of the total number of *Gfp*^+^ and *Crhr1*^+^ nuclei in the ROI (1024 × 10^24^; 40×) in the LA of 11 images from 3 mice. ***C***, Graph of the percentage of nuclei coexpressing *Crhr1* in *Gfp*^+^ nuclei (black bar), and the percentage of nuclei coexpressing *Gfp* in *Crhr1*^+^ nuclei (white bar). ***D–F***, Representative images in the LA are shown for *Crhr1* and DAPI (***D***); *Slc17a7* and DAPI (***E***); and the merged imaged of *Crhr1*, *Slc17a7*, and DAPI (***F***; *Crhr1 *=* *red fluorescence, *Slc17a7 *=* *green fluorescence, and DAPI = blue fluorescence). Scale bar, 10 μm. ***G***, Summary of the total number of *Crhr1^+^* and *Slc17a7^+^* nuclei in the ROI (1024 × 10^24^; 40×) in the LA of 10 images from 3 CRF1:GFP mice. ***H***, Graph of the percent of nuclei coexpressing *Crhr1* in *Slc17a7^+^* nuclei (black bar) and nuclei coexpressing *Slc17a7* in *Crhr1^+^* (white bar).

To validate the fidelity of the CRF1:GFP expression in the LA, we used the RNAscope assay (*n* = 11 images from three mice) to examine colocalization of *Crhr1*, the transcript for CRF1, and *Gfp*, the transcript for green fluorescent protein (Fig. 1*A*). The number of positive nuclei in the ROI was consistent between groups (Fig. 1*B*). Approximately 74% of *Crhr1^+^* neurons coexpress *Gfp* and 84% of *Gfp^+^* neurons coexpress *Crhr1* (Fig. 1*C*), indicating substantial penetrance and fidelity, respectively. To identify the phenotype of CRF1^+^ neurons in the LA, we performed *in situ* hybridization in brain sections from CRF1:GFP mice (*n* = 10 images from three mice) to examine colocalization of *Crhr1* and *Slc17a7*, the transcript for VGluT1. Consistent with GFP expression and the established glutamatergic makeup of the BLA, *Crhr1* and *Slc17a7* were similarly expressed in the LA (Fig. 1*D****–****F*). The number of positive nuclei counted in the ROI was not significantly different between *Slc17a7^+^* and *Crhr1^+^* (Fig. 1*G*). Approximately 60% of *Slc17a7^+^* neurons coexpress *Crhr1*, and ∼80% of *Crhr1^+^* neurons coexpress *Slc17a7* (Fig. 1*H*). These data suggest that *Crhr1^+^* neurons make up a subpopulation of LA glutamatergic cells and that the majority of *Crhr1^+^* LA neurons are glutamatergic.

**Figure 2.**
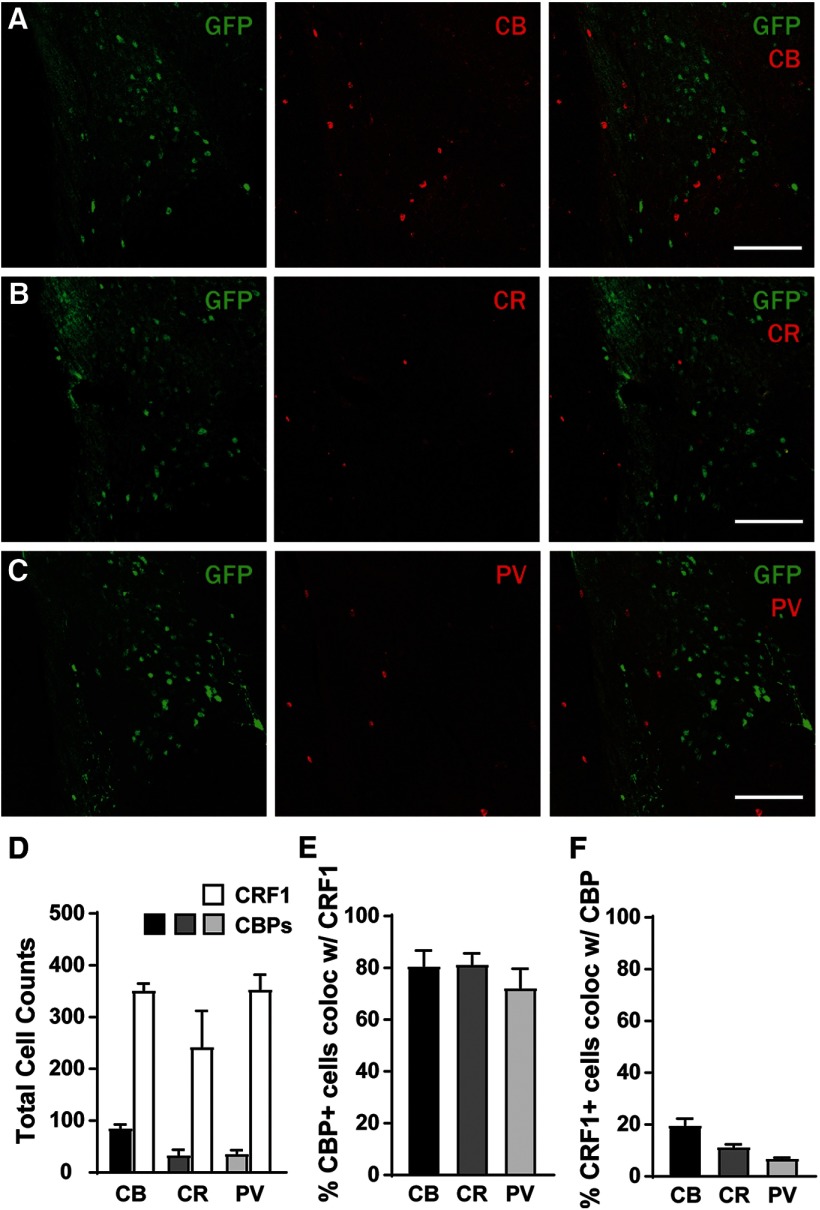
Calcium binding protein expression in CRF1^+^ and CRF1^−^ lateral amygdala neurons. ***A***, Photomicrograph (10×) of GFP expression (green fluorescence, left), calbindin expression (red fluorescence, center), and merge (right). Scale bar, 100 μm. ***B***, Photomicrograph (10×) of GFP expression (green fluorescence, left), calretinin expression (red fluorescence, center), and merge (right). Scale bar, 100 μm. ***C***, Photomicrograph (10×) of GFP expression (green fluorescence, left), parvalbumin expression (red fluorescence, middle), and merge (right). Scale bar, 100 μm. ***D***, Summary of total cells expressing CRF1 (GFP) and CBPs, *n* = 16 sections from 4 mice. ***E***, Percentage of CBP^+^ cells that coexpress CRF1. ***F***, Percentage of CRF1^+^ cells that coexpress CBPs.

The LA is composed of glutamatergic projection neurons as well as local GABAergic interneurons (Sosulina et al., 2006). The results of the *in situ* experiments indicated that a subpopulation of the CRF1^+^ neurons of the LA do not express *Slc17a7*, suggesting that these neurons are not glutamatergic but may express calcium binding proteins (CBPs) associated with GABAergic interneurons. Work by Calakos et al. (2017) reported that the majority of PV-containing neurons in the BLA also expressed CRF1, but the expression of CBPs in CRF1^+^ neurons of the LA is unknown. We examined PV and GFP colocalization in the LA of CRF1:GFP mice (*n* = 16 sections from four mice) as well as calbindin (CB) and calretinin (CR). For the purpose of clarity, we refer to GFP^+^ and GFP^−^ neurons throughout as CRF1^+^ and CRF1^−^, respectively. We observed expression of CB, CR, and PV interspersed with GFP in the LA (Fig. 2*A–C*), but there were more CRF1^+^ cells than CBP-containing cells (Fig. 2*D*). Consistent with Calakos et al. (2017), we observed that a substantial percentage (∼80%) of CBP^+^ cells also expressed GFP (Fig. 2*E*), suggesting that the majority of LA neurons that express CBPs also contain CRF1. However, the percentage of CRF1^+^ neurons that contain CBPs was much lower (<20%; Fig. 2*F*), suggesting that the majority of CRF1^+^ neurons are likely not interneurons that express these calcium binding proteins. Together, the results of the *in situ* and immunohistochemistry experiments identify the CRF1^+^ neurons of the LA as a mostly (∼80%) glutamatergic population with a smaller (∼20%) population of neurons that express CBPs (potentially GABAergic interneurons).

### Membrane properties and excitability

**Figure 3.**
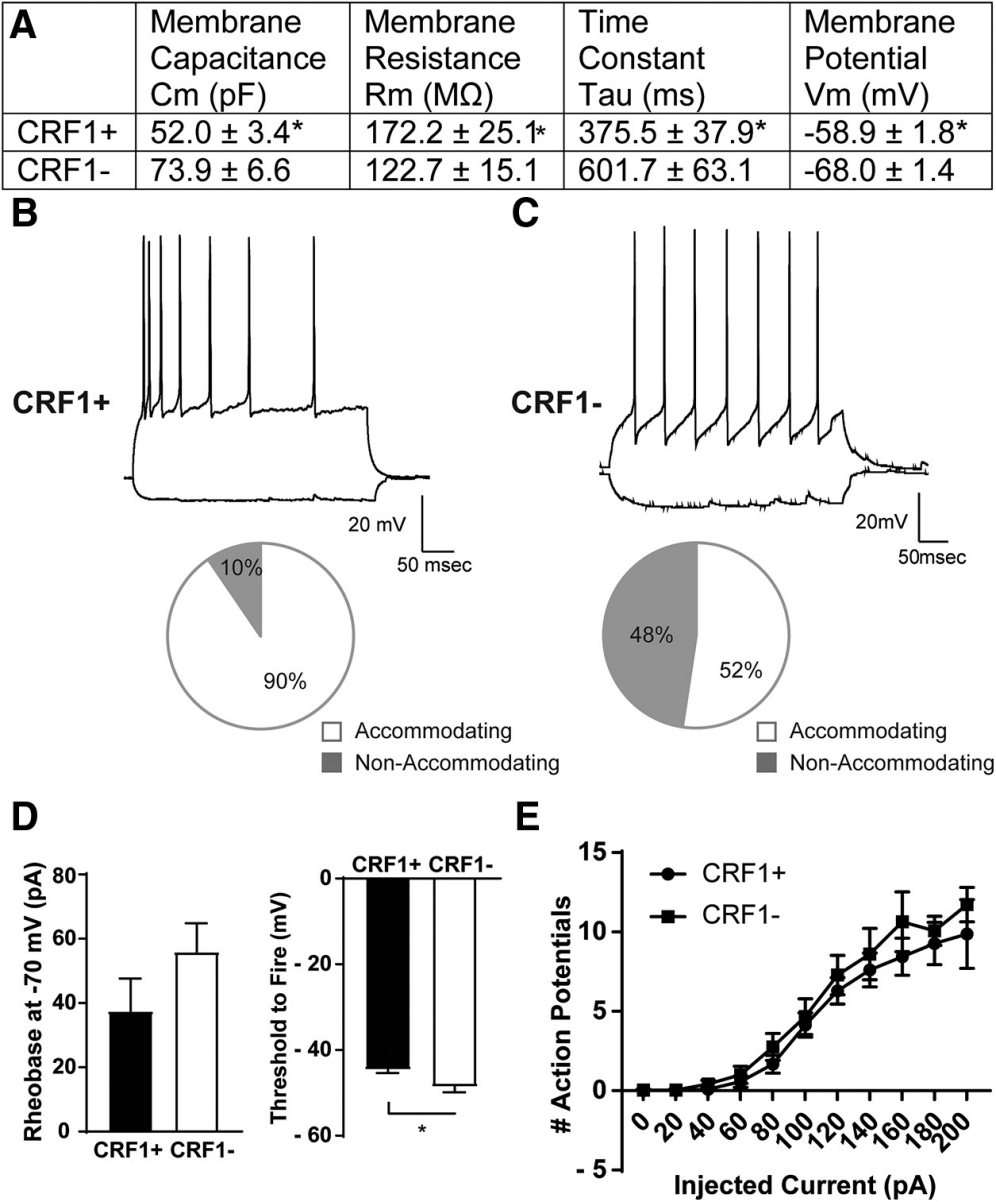
Membrane characteristics and excitability of CRF1^+^ and CRF1^−^ lateral amygdala neurons. ***A***, Summary of membrane characteristics of CRF1^+^ (*n* = 28) and CRF1^−^ (*n* = 28) LA cells. ***B***, Representative current-clamp recording of LA CRF1^+^ neuron action potentials elicited by 100 pA current injection (top) and the relative proportion of CRF1^+^ LA neurons displaying spike accommodation with current injection (bottom). ***C***, Representative current-clamp recording of LA CRF1^−^ neuron action potentials elicited by 100 pA current injection (top) and the relative proportion of CRF1^−^ LA neurons displaying spike accommodation with current injection (bottom). ***D***, Summary of rheobase at −70 mV (left) and the threshold to fire (right) of CRF1^+^ and CRF1^−^ LA neurons. *p <* *0.05 by unpaired *t* test comparing CRF1^+^ to CRF1^−^ cells. ***E***, Summary of action potentials by current injection in CRF1^+^ and CRF1^−^ LA neurons.

LA neurons were identified and targeted for electrophysiological recording based on GFP expression. CRF1^+^ neurons (*n* = 28 cells from 14 mice) possessed a significantly smaller membrane capacitance (*t*_(54)_ = 2.96; *p *=* *0.0046 by unpaired *t* test, 21.84 ± 7.39 pF effect size; 95% confidence interval, −36.65 to −7.02), increased membrane resistance (*t*_(30)_ = 2.34; *p* = 0.0260 by unpaired *t* test; 99.46 ± 42.48 mV effect size; 95% confidence interval, −186.2 to −12.71), lower time constant (*t*_(54)_ = 3.08; *p *=* *0.0033 by unpaired *t* test; 226 ± 73.56 ms effect size; 95% confidence interval, −373.6 to −78.69), and higher resting membrane potential (*t*_(54)_ = 3.95; *p *=* *0.0002 by unpaired *t* test; 9.114 ± 2.31 mV effect size; 95% confidence interval, 4.49–13.74) compared with CRF1^−^ neurons (*n* = 28 cells from 15 mice; Fig. 3*A*). Whole-cell current-clamp recordings and a step protocol consisting of hyperpolarizing (−60 pA) to depolarizing (100 pA; Fig. 3*B*,*C*) current injections were used to examine the spiking properties of CRF1^+^ and CRF1^−^ LA neurons. The large majority (90%) of CRF1^+^ neurons exhibited spike accommodation (Fig. 3*B*, bottom), whereas CRF1^−^ neurons were more variable (52% accommodating; Fig. 3*C*, bottom). We observed no significant differences in rheobase between CRF1^+^ (37.52 ± 10.11 pA) and CRF1^−^ neurons (55.94 ± 8.90 pA; Fig. 3*D*, left); however, we did observe a significantly lower threshold to fire in CRF1^−^ neurons (−48.64 ± 1.25 pA) versus CRF1^+^ neurons (−44.61 ±0.78 pA; *t*_(40)_ = 2.73; *p *=* *0.0093; effect size, 4.03 ± 1.48 pA; 95% confidence interval, −7.01 to −1.048; Fig. 3*D*, right). In addition, we found no differences in action potentials elicited by ascending current injection between CRF1^+^ and CRF1^−^ neurons (Fig. 3*E*).

### Phasic and tonic inhibitory transmission

**Figure 4.**
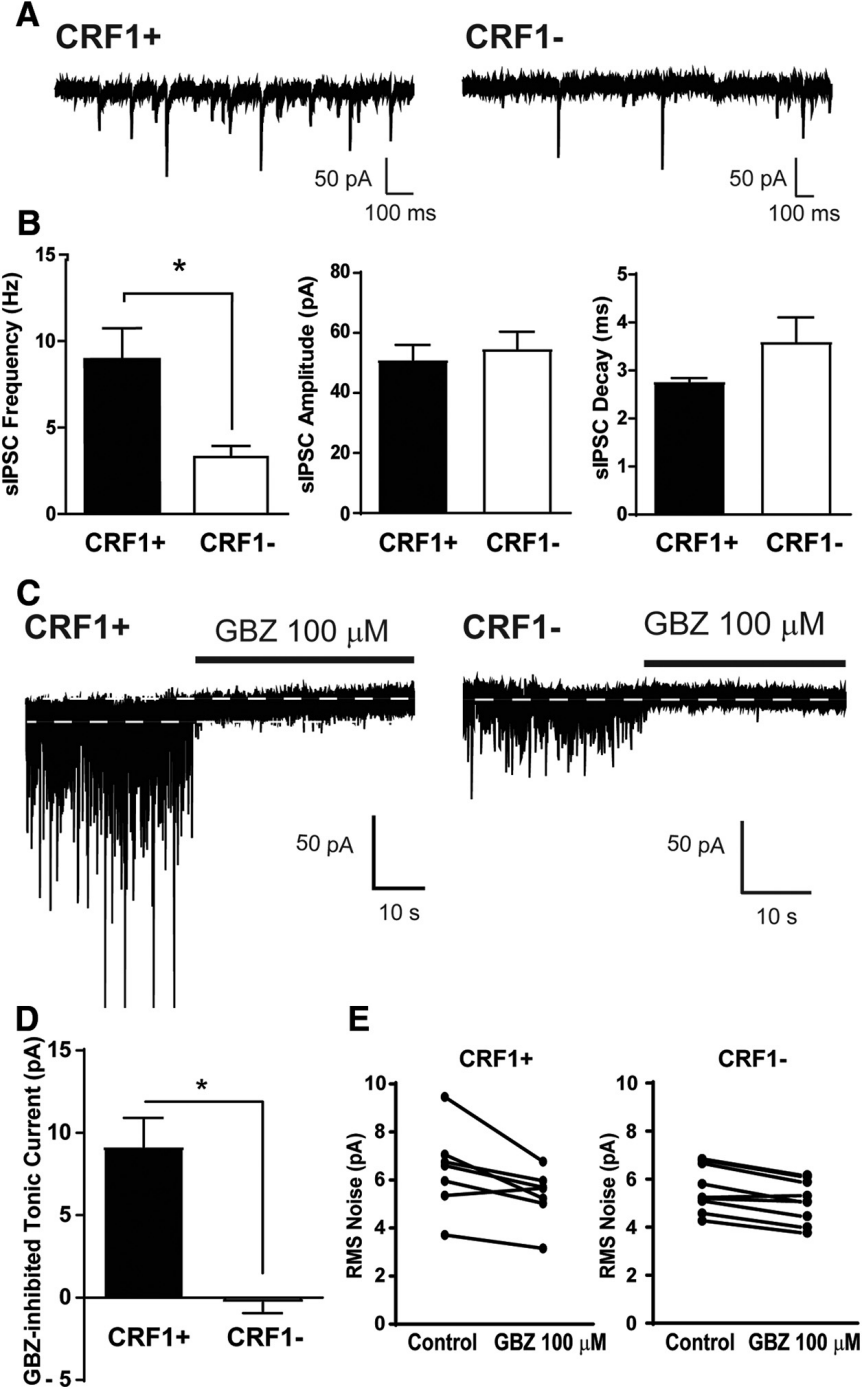
Phasic and tonic inhibitory transmission in CRF1 lateral amygdala neurons. ***A***, Representative voltage-clamp recording of a CRF1^+^ cell (left) and a CRF1^−^ cell (right). ***B***, Summary of sIPSC frequency (left), amplitude (center), and decay (right) of CRF1^+^ and CRF1^−^ cells. *p <* *0.05 by unpaired *t* test comparing CRF1^+^ to CRF1− cells. ***C***, Representative voltage-clamp recording of a CRF1^+^ cell (left) and a CRF1^−^ cell (right) during GBZ superfusion (100 μm). White dashed line indicates level of holding current before and after GBZ superfusion. ***D***, Summary of the tonic current revealed by gabazine. *p <* *0.05 by unpaired *t* test comparing CRF1^+^ to CRF1^−^ cells. ***E***, Summary of the change in rms noise induced by gabazine superfusion in CRF1^+^ (left) and CRF1^−^ (right) cells.

Whole-cell voltage-clamp recordings of sIPSCs were performed to assess baseline phasic inhibitory transmission. CRF1^+^ neurons had a significantly higher average baseline sIPSC frequency (9.0 ± 1.8 Hz; *n* = 7 cells from six mice; Fig. 4A,*B*) compared with CRF1^−^ neurons (3.3 ± 0.6 Hz; *t*_(14)_ = 3.30; *p* = 0.0053 by unpaired *t* test; 5.71 ± 1.73 Hz effect size with 95% confidence interval of 2.00–9.42; *n* = 9 cells from five mice; Fig. 4*A*,*B*), and no difference in sIPSC amplitude (51.01 ± 5.0 and 54.7 ±5.7 pA; *p* = 0.64; Fig. 4*A*,*B*), decay (2.77 ± 0.08 and 3.60 ±0.5 ms; *p* = 0.17; Fig. 4*A*,*B*), or rise time (1.61 ± 0.12 and 1.58 ± 0.16 ms; *p* = 0.88) between CRF1^+^ and CRF1^−^ LA neurons, respectively.

We assessed tonic conductance in CRF1^+^ (*n* = 7 cells from five mice) and CRF1^−^ (*n* = 9 cells from five mice) LA neurons using whole-cell voltage-clamp recordings. The basal holding current was −28.32 ± 20.67 pA in CRF1^+^ neurons and −17.19 ± 14.65 pA in CRF1^−^ neurons. A GABA_A_ receptor-mediated tonic current was defined as the difference in holding current (i.e., the current required to maintain the neuron at −60 mV) before and after application of a GABA_A_ receptor antagonist. Focal application of the GABA_A_ receptor antagonist GBZ (100 μm) produced a significant reduction in holding current in CRF1^+^ neurons (9.2 ± 1.8 pA, *n* = 7; Fig. 4*C*, left trace, 4*D*; *t*_(14)_ = 5.56, *p *=* *0.002 by one-sample *t* test; 9.45 ± 1.70 pA effect size; 95% confidence interval, 5.81–13.09) and a reduction in the amplitude of the holding current or root mean square (rms) noise (6.4 ± 0.7-5.4 ± 0.4 pA; Fig. 4*E*, left; *t*_(6)_ = 2.93, *p *=* *0.0264 by paired *t* test; 1.06 ± 0.364 pA effect size; 95% confidence interval, 0.17–1.94). In CRF1^−^ neurons, focal application of GBZ (100 μm) produced no change in holding current (−0.3 ± 0.6 pA, *n* = 9; Fig. 4*C*, right trace, *D*; *p *=* *0.6568 by one-sample *t* test) and a reduction in rms noise of a much smaller magnitude (5.6 ± 0.3–5.1 ± 0.3 pA; Fig. 4*E*, right; *t*_(8)_ = 5.24, *p *=* *0.0008 by paired *t* test; 0.51 ± 0.10 pA effect size; 95% confidence interval, 0.29–0.74). The reduction in holding current was significantly greater in CRF1^+^ neurons compared with CRF1^−^ neurons (Fig. 4*D*; *t*_(14)_ = 5.56, *p *=* *0.0001 by unpaired *t* test; 9.45 ± 1.70 pA effect size; 95% confidence interval, 5.81–13.09).

### Expression of GABA_A_ receptor subunits

**Figure 5.**
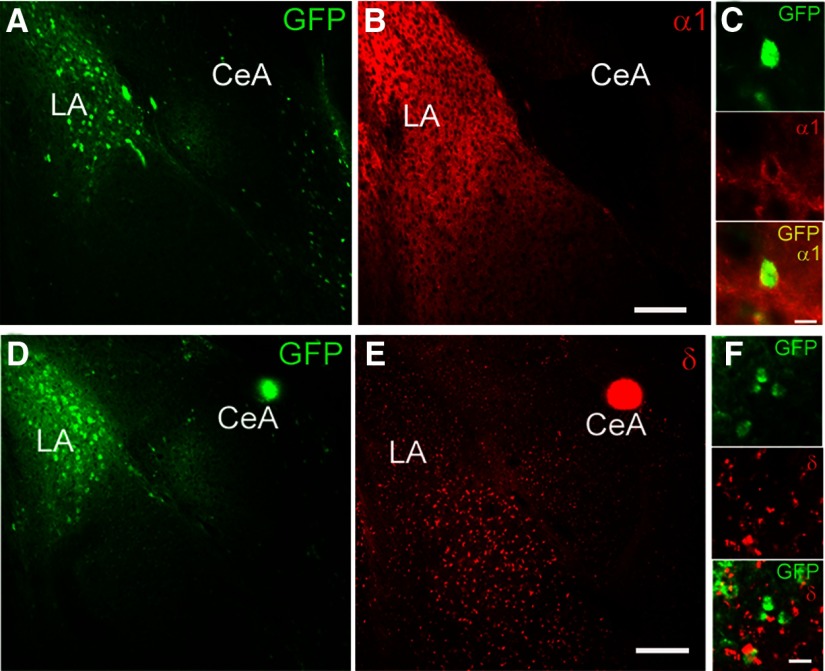
GABA_A_ subunit expression in CRF1^+^ and CRF1^−^ lateral amygdala neurons. ***A***, Photomicrograph (10×) of GFP expression (green fluorescence) in LA. ***B***, Photomicrograph (10×) of α1 GABA_A_ receptor subunit expression (red fluorescence) in LA. Scale bar, 100 μm. ***C***, Photomicrograph (60×) of GFP expression (top), α1 expression (center), and merge (bottom) in LA highlighting a single cell exhibiting coexpression of GFP and α1. Scale bar, 10 μm. ***D***, Photomicrograph (10×) of GFP expression (green fluorescence) in LA. ***E***, Photomicrograph (10×) of δ GABA_A_ receptor subunit expression (red fluorescence) in LA. Scale bar, 100 μm. ***F***, Photomicrograph (60×) of GFP expression (top), δ expression (center), and merge (bottom) in LA. Scale bar, 10 μm.

The phasic and tonic conductance of GABA_A_ receptors is dependent on specific subunit configurations and/or expression. We performed double-label immunohistochemical studies examining α1 and δ GABA_A_ receptor subunit expression in CRF1^+^ and CRF1^−^ neurons in the LA (*n* = 12 sections from four mice). The LA contains a significant number of CRF1^+^ cells, in contrast with sparse GFP expression in the BLA (Fig. 5*A*,*D*). The α1 GABA_A_ receptor subunit has dense expression in the LA (Fig. 5*B*) and displays colocalization with GFP (Fig. 5*C*), indicating expression in the majority of CRF1^+^ neurons. In contrast, δ GABA_A_ receptor subunit expression was greater in the body of the BLA than in the LA (Fig. 5*E*) and displays minimal colocalization with GFP (Fig. 5*F*), indicating little to no expression in CRF1^+^ neurons in the LA.

**Figure 6.**
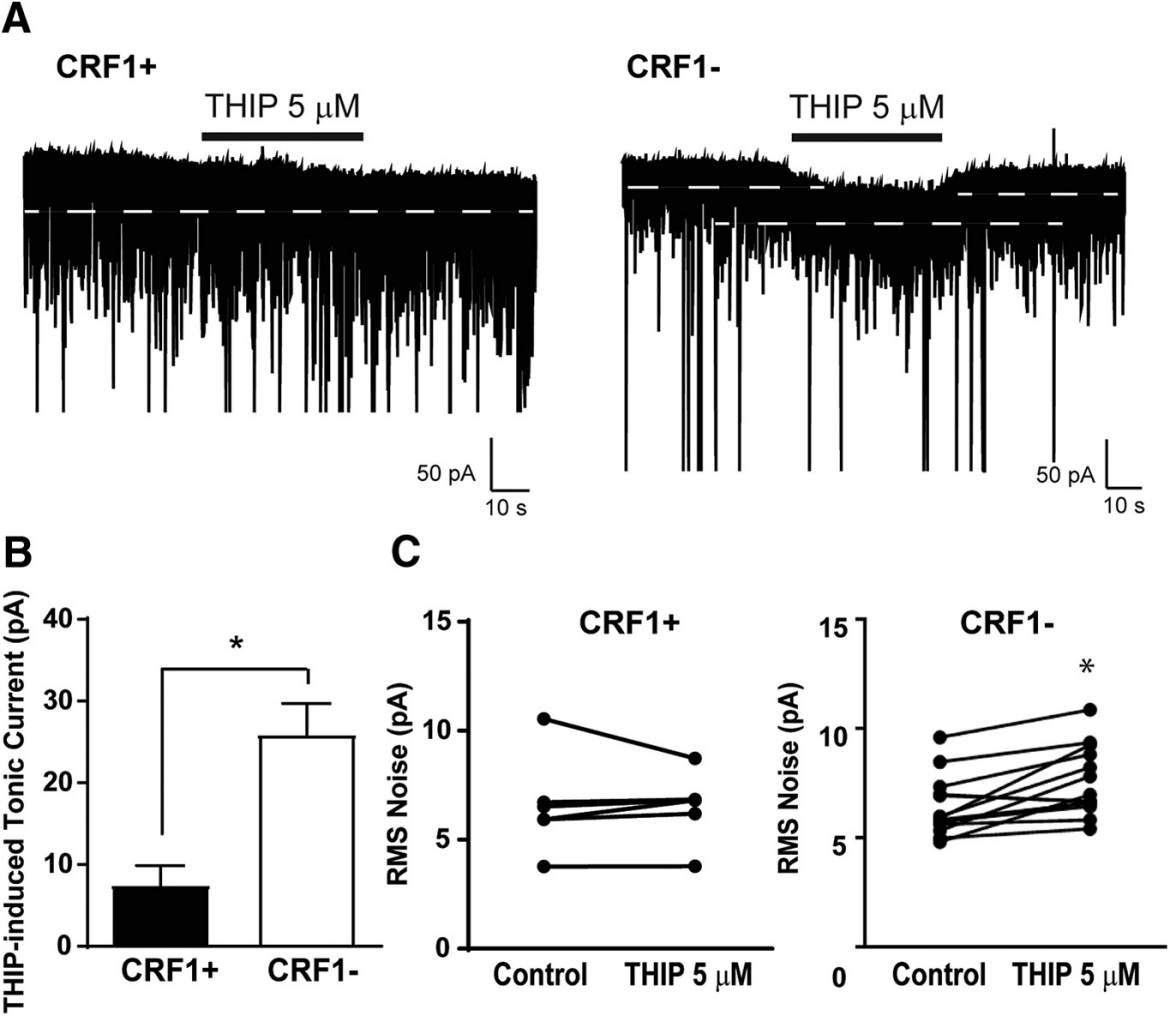
Contribution of δ subunit-containing GABA_A_ receptors to tonic conductance in CRF1^+^ and CRF1^−^ lateral amygdala neurons. ***A***, Representative voltage-clamp recording of a CRF1^+^ (left) and CRF1^−^ (right) cell during superfusion of the δ subunit-preferring GABA_A_ agonist THIP (5 μm). White dashed line indicates level of holding current before and after THIP superfusion. ***B***, Summary of the tonic current induced by THIP in CRF1^+^ and CRF1^−^ cells; *p <* *0.05 by unpaired *t* test comparing CRF1^+^ to CRF1− cells. ***C***, Summary of the change in rms noise induced by THIP superfusion in CRF1^+^ (left) and CRF1^−^ (right) cells. *p < 0.05 by paired *t* test comparing differences between control and THIP 5 μm

The δ subunit is associated with tonic conductance in a number of brain areas, including the hippocampus, cerebellum, cortex, and amygdala (Saxena and Macdonald, 1996; Stell et al., 2003; Krook-Magnuson and Huntsman, 2005). Thus, we examined the functional contribution of δ subunit-containing GABA_A_ receptors in the LA using the δ subunit-preferring agonist THIP (5 μm). Focal application of THIP produced a modest increase in holding current in CRF1^+^ neurons (7.5 ± 2.4 pA; *n* = 6 cells from 6 mice; *t*_(5)_ = 3.12, *p* =0.0262 by one-sample *t* test; Fig. 6*A*, left, *B*) and CRF1^−^ neurons (25.9 ± 3.8 pA; *n* = 14 cells from 10 mice; *t*_(13)_ = 6.82, *p *<* *0.001 by one-sample *t* test; Fig. 6*A*, right trace, *B*). This increase was significantly greater in CRF1^−^ neurons compared with CRF1^+^ neurons (*t*_(18)_ = 3.03, *p =* *0.0072 by unpaired *t* test; 18.44 ± 6.09 pA effect size; 95% confidence interval, −31.24 to −5.65). Consistent with the observed effects on holding current, focal application of THIP onto CRF1^+^ neurons resulted in no change in the amplitude of the holding current or rms noise (6.6 ± 0.9–6.5 ± 0.7 pA; Fig. 6*C*, left; *p *=* *0.9183 by paired *t* test) but significantly increased rms noise in CRF1^−^ neurons (6.4 ± 0.4–7.5 ± 0.4 pA; Fig. 6*C*, right; *t*_(13)_ = 4.03, *p *=0.0014 by paired *t* test; 1.16 ± 0.29 pA effect size; 95% confidence interval, −1.78 to −0.54). Together, these findings indicate that the δ subunit is expressed predominantly in CRF1^−^ neurons, whereas the α1 subunit is expressed predominantly in CRF1^+^ neurons, and that δ-containing GABA_A_ receptors contribute to tonic conductance in CRF1^−^ but not CRF1^+^ neurons.

### Acute cellular ethanol exposure

**Figure 7.**
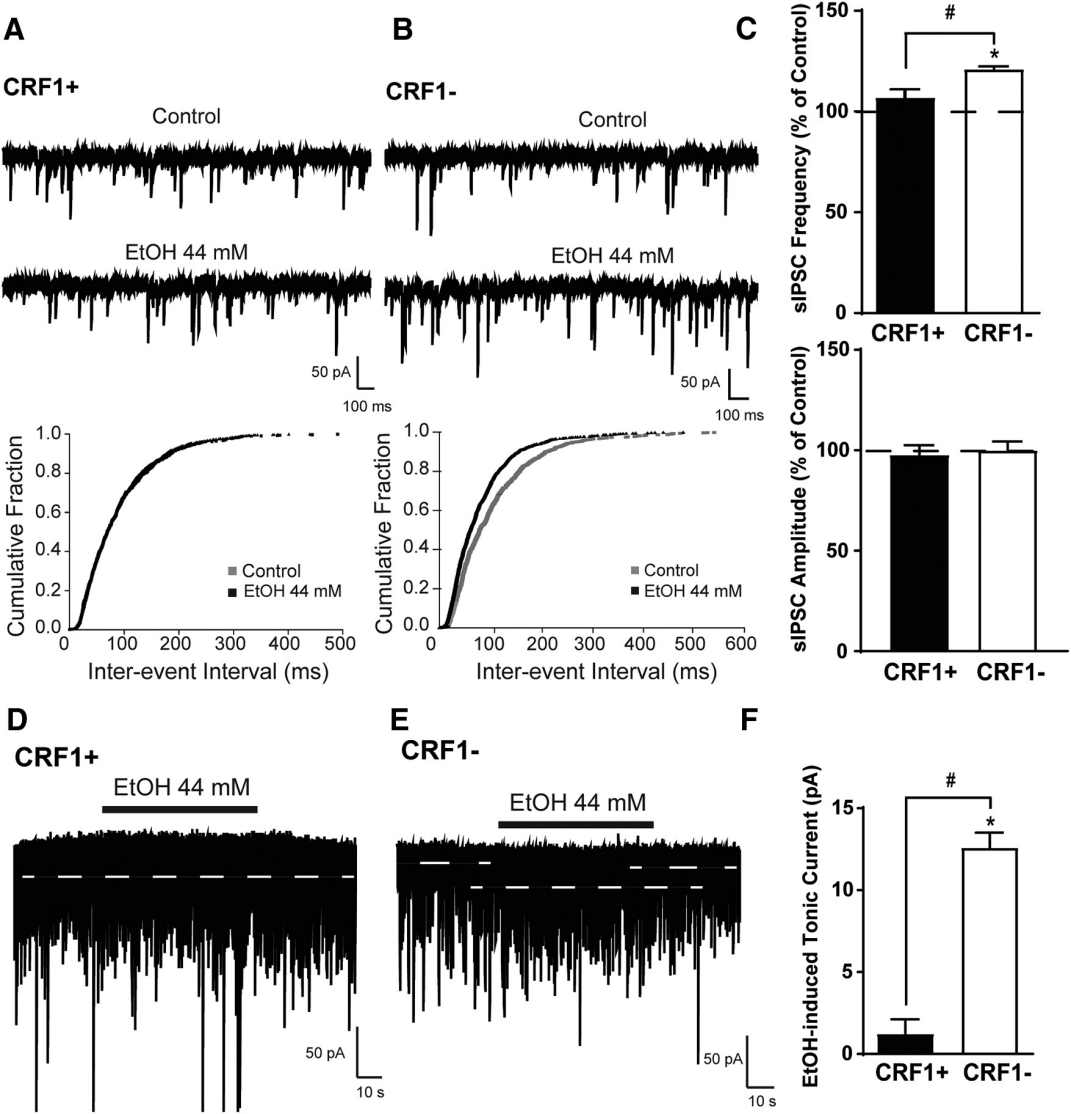
Effects of acute ethanol exposure on phasic and tonic inhibitory transmission in CRF1^+^ and CRF1^−^ lateral amygdala neurons. ***A***, Representative voltage-clamp recording (top) and cumulative probability histogram of interevent interval (bottom) of a CRF1^+^ cell during superfusion of EtOH (44 mm). ***B***, Representative voltage-clamp recording (top) and cumulative probability histogram of interevent interval (bottom) of a CRF1^−^ cell during superfusion of EtOH (44 mm). ***C***, Summary of the change in sIPSC frequency (top) and amplitude (bottom) following ethanol superfusion compared with baseline in CRF1^+^ and CRF1^−^ cells. *p <* *0.05 by one-sample *t* test comparing differences from baseline within cell type; #*p *<* *0.05 by unpaired *t* test comparing CRF1^+^ to CRF1^−^ cells. ***D***, Representative voltage-clamp recording of a CRF1^+^ cell during superfusion of EtOH (44 mm). White dashed line indicates the level of holding current before and after EtOH superfusion. ***E***, Representative voltage-clamp recording of a CRF1^−^ cell during superfusion of EtOH (44 mm). White dashed line indicates the level of holding current before and after EtOH superfusion. ***F***, Summary of the tonic current induced by ethanol in CRF1^+^ and CRF1^−^ cells. *p <* *0.05 by one-sample *t* test comparing differences from baseline within cell type; #*p *<* *0.05 by unpaired *t* test comparing CRF1^+^ to CRF1^−^ cells.

GABA_A_ receptors are sensitive to ethanol, and tonic conductance has been shown to be selectively augmented by acute ethanol (Wallner et al., 2003; Wei et al., 2004; Herman et al., 2013). Focal application of EtOH (44 mm) did not significantly alter sIPSC interevent interval or sIPSC frequency (107 ± 4.0% of control; *p *=* *0.1514 by one-sample *t* test; *n* = 5 cells from five mice; Fig. 7*A*,*C*, top) in CRF1^+^ neurons, but decreased interevent interval and increased sIPSC frequency in CRF1^−^ neurons (121.1 ± 1.3% of control; *n* = 5 cells from five mice; Fig. 7*B*,*C*, top; *t*_(3)_ = 15.82, *p =* *0.0005 by one-sample *t* test; *t*_(7)_ = 3.03, #*p *=* *0.01,915 by unpaired *t* test; 14.08 ± 4.65% control effect size; 95% confidence interval, −25.08 to −3.09). Ethanol did not change sIPSC amplitude (97.76 ± 4.8% and 100.1 ± 4.3% of control, *p *=* *0.7285 by unpaired *t* test; Fig. 7*C*, bottom), rise (105.0 ± 5.8% and 105.6 ± 2.1% of control, *p *=* *0.9222 by unpaired *t* test), or decay (104.5 ± 2.8% and 103.3 ± 2.2% of control, *p *=* *0.7395 by unpaired *t* test) in CRF1^+^ or CRF1^−^ neurons, respectively. Additionally, focal application of ethanol did not significantly change the holding current of CRF1^+^ neurons (1.2 ± 0.9 pA, *n* = 5 cells from five mice; Fig. 7*D*,*F*; *p *=* *0.2304 by one-sample *t* test), but did significantly increase holding current in CRF1^−^ neurons (12.6 ± 0.9 pA, *t*_(4)_ = 14.11, *p =* *0.0001 by one-sample *t* test, *n* = 5 cells from five mice; *t*_(8)_ = 9.09, #*p *=* *0.0001 by unpaired *t* test; 11.37 ± 1.25 pA effect size; 95% confidence interval, −14.26 to −8.487; Fig. 7*E*,*F*). Acute ethanol did not significantly affect rms noise in CRF1^+^ neurons (5.9 ± 0.2 pA at baseline to 6.1 ± 0.4 pA after EtOH, *p *=* *0.3868) or CRF1^−^ neurons (6.3 ± 0.5 pA at baseline to 6.2 ± 0.5 pA after EtOH, *p *=* *0.6131).

### Chronic intermittent ethanol exposure

**Figure 8.**
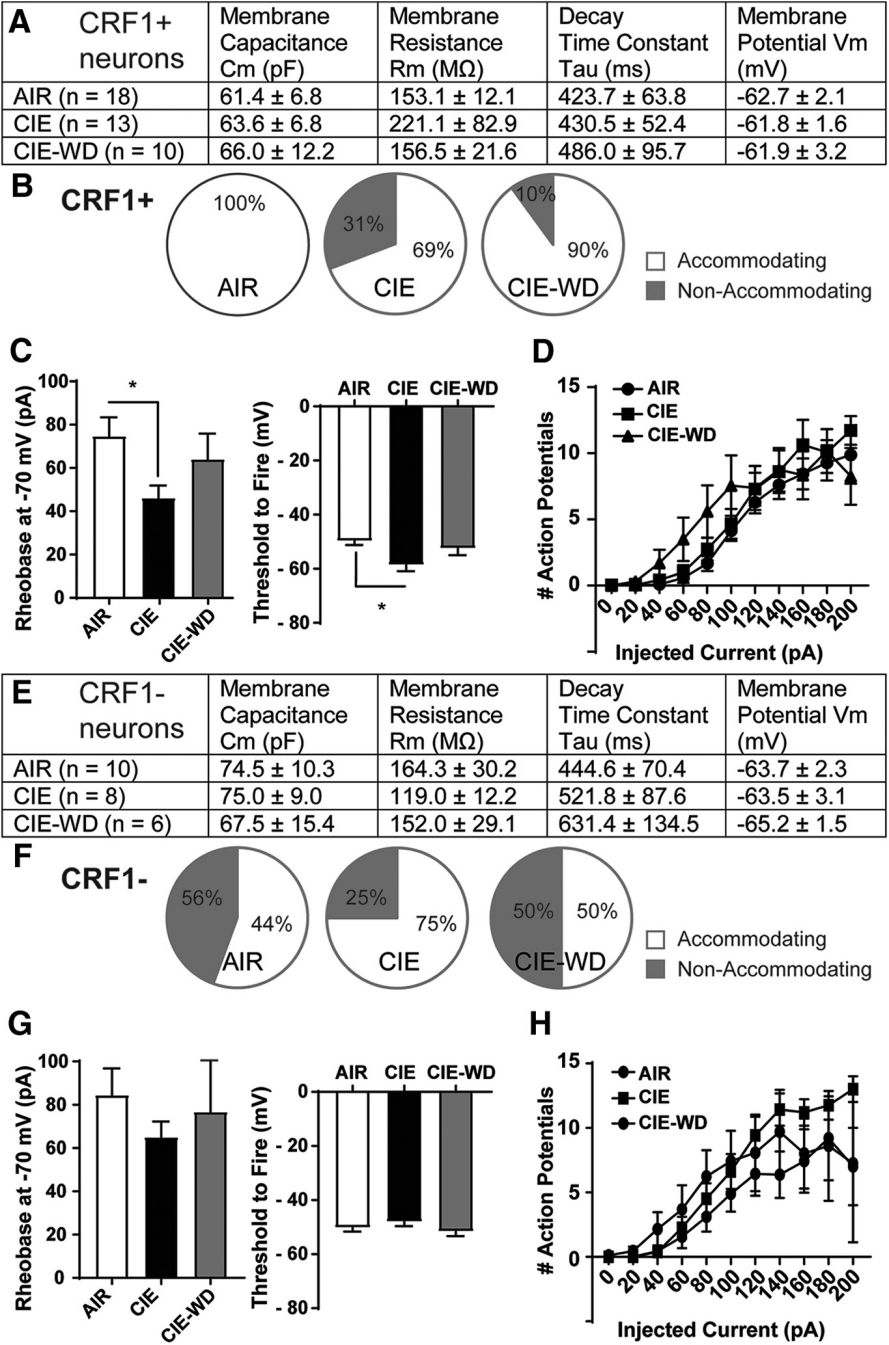
Effects of chronic ethanol vapor on membrane characteristics and excitability in CRF1^+^ and CRF1^−^ lateral amygdala neurons. ***A***, Summary of membrane characteristics of CRF1^+^ LA neurons from AIR, CIE, and CIE-WD mice. ***B***, Relative proportion of CRF1^+^ neurons exhibiting spike accommodation from AIR (left), CIE (center), and CIE-WD (right) mice. ***C***, Summary of rheobase at −70 mV (left) and the threshold to fire (right) of CRF1^+^ neurons from AIR, CIE, and CIE-WD mice. *p <* *0.05 by unpaired *t* test comparing CRF1^+^ neurons from AIR mice to CRF1^+^ neurons from CIE mice. ***D***, Summary of action potentials by current injection in CRF1^+^ neurons from AIR, CIE, and CIE-WD mice. ***E***, Summary of membrane characteristics of CRF1^−^ LA neurons from AIR, CIE, and CIE-WD mice. ***F***, Relative proportion of CRF1^−^ neurons exhibiting spike accommodation from AIR (left), CIE (middle), and CIE-WD (right) mice. ***G***, Summary of rheobase at −70 mV (left) and the threshold to fire (right) of CRF1^−^ neurons from AIR, CIE, and CIE-WD mice. ***H***, Summary of action potentials by current injection in CRF1^−^ neurons from AIR, CIE, and CIE-WD mice.

To examine the sensitivity of LA neurons to chronic ethanol exposure, we subjected CRF1:GFP mice to CIE vapor exposure (4–5 weeks) and CIE followed by 3–7 d of withdrawal (CIE-WD). There were no significant changes in membrane properties in CRF1^+^ neurons following ethanol vapor exposure or withdrawal (Fig. 8*A*), and, consistent with naive neurons, the majority of CRF1^+^ neurons from AIR, CIE, and CIE-WD mice exhibited spike accommodation (Fig. 8*B*). Rheobase was reduced in CRF1^+^ neurons from CIE mice (74.74 ± 8.63 pA, *n* = 19 cells from 11 mice) compared with neurons from AIR mice (46.15 ±5.72 pA, *n* = 19 cells from 6 mice; *t*_(30)_ = 2.49, *p *=* *0.0187 by unpaired *t* test; effect size, −28.58 ± 11.49 pA; 95% confidence interval, −52.05 to −5.113; Fig. 8*C*, left). Rheobase did not significantly differ between CRF1^+^ neurons from CIE-WD mice (64 ± 11.85 pA, *n* = 10 cells from three mice) and neurons from AIR mice (*p *=* *0.4708; Fig. 8*C*, left). The threshold to fire was also reduced in neurons from CIE mice (−58.58 ± 2.35 mV) versus neurons from AIR mice (−49.71 ± 1.50 mV; *t*_(30)_ = 3.34, *p *=* *0.0022 via unpaired *t* test; effect size, 8.87 ± 2.65 mV; 95% confidence intervals, −14.29 to −3.46; Fig. 8*C*, right) but was not different in neurons from CIE-WD mice (−52.43 ± 2.55 mV) versus neurons from AIR mice (*p *=* *0.3353). In addition, we found no differences in action potentials elicited by ascending current injection among the three exposure conditions (Fig. 8*D*). Together, these findings indicate increases in the excitability of LA CRF1^+^ neurons following CIE exposure that are normalized under withdrawal conditions.

There were no significant changes in membrane properties in CRF1^−^ neurons following ethanol vapor exposure or withdrawal (Fig. 8*E*), and, consistent with neurons from naive mice, approximately half of CRF1^−^ neurons from AIR and CIE-WD mice exhibited spike accommodation (Fig. 8*F*). No changes in rheobase were observed among CRF1^−^ neurons from AIR mice (84.44 ± 12.37 pA, *n* = 9 cells from four mice), CIE mice (65.00 ± 7.32 pA, *n* = 8 cells from three mice), or CIE-WD mice (76.67 ± 26.03 pA, *n* = 6 cells from three mice; Fig. 8*G*, left). The threshold to fire was also comparable in CRF1^−^ neurons from AIR mice (−50.18 ± 1.47 mV) versus neurons from CIE mice (−48.00 ± 1.62 mV) and CIE-WD mice (−51.55 ± 1.76 mV; Fig. 8*G*, right). No significant differences in the number of action potentials across current injection steps emerged among CRF1^−^ neurons from AIR, CIE, or CIE-WD mice (Fig. 8*H*). These findings indicate no changes in the excitability of CRF1^−^ neurons following AIR, CIE, or CIE-WD exposure.

**Figure 9.**
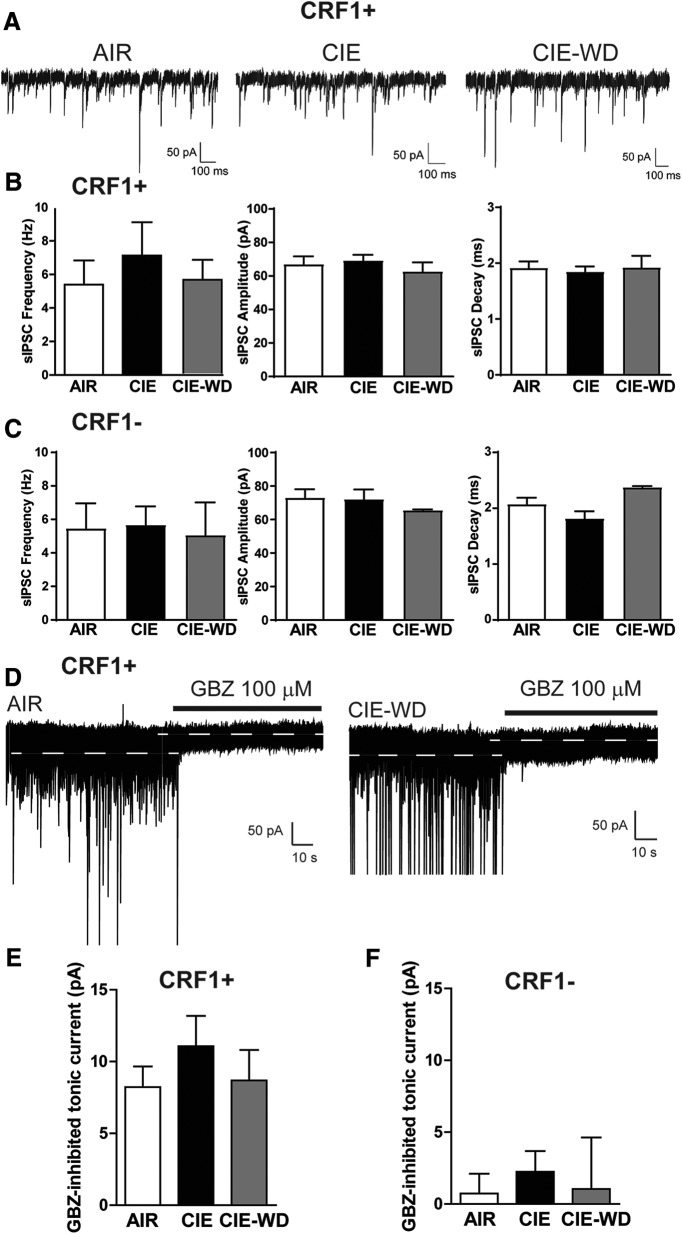
Effects of chronic ethanol vapor on phasic and tonic inhibitory transmission in CRF1^+^ and CRF1^−^ lateral amygdala neurons. ***A***, Representative voltage-clamp recordings of CRF1^+^ neurons from AIR (left), CIE (center), and CIE-WD (right) mice. ***B***, Summary of sIPSC frequency (left), amplitude (middle), and decay (right) in CRF1^+^ neurons from AIR, CIE, and CIE-WD mice. ***C***, Summary of sIPSC frequency (left), amplitude (center), and decay (right) in CRF1^−^ neurons from AIR, CIE, and CIE-WD mice. ***D***, Representative voltage-clamp recording of CRF1^+^ cells from AIR (left) and CIE-WD (right) mice during GBZ superfusion (100 μm). White dashed line indicates the level of holding current before and after GBZ superfusion. ***E***, Summary of tonic current revealed by gabazine superfusion in CRF1^+^ cells. ***F***, Summary of tonic current revealed by gabazine superfusion in CRF1^−^ cells.

We next assessed phasic inhibitory transmission in CRF1^+^ and CRF1^−^ LA neurons following vapor exposure. There were no significant changes in sIPSC frequency (5.4 ± 1.4, 7.1 ± 2.0, and 5.6 ± 1.2 Hz; *p *=* *0.7112 by one-way ANOVA; *n* = 5–8 cells from 3–4 mice/group; Fig. 9*A*,*B*, left), sIPSC amplitude (67.0 ± 4.7, 69.2 ± 3.4, and 62.7 ± 5.5 pA; *p *=* *0.6551 by one-way ANOVA; *n* = 5–8 cells from 3–4 mice/group; Fig. 9*A*,*B*, middle), sIPSC rise (1.9 ± 1.1, 1.8 ± 0.1, and 1.9 ± 0.2 ms; *p *=* *0.8369 by one-way ANOVA; *n* = 5–8 cells from 3–4 mice/group; Fig. 9*A*), or sIPSC decay (1.9 ± 1.1, 1.8 ± 0.1, and 1.9 ± 0.2 ms; *p *=* *0.9120 by one-way ANOVA; *n* = 5–8 cells from 3–4 mice/group; Fig. 9*A*,*B*, right) in CRF1^+^ neurons from AIR, CIE, and CIE-WD mice, respectively. CRF1^−^ neurons from AIR, CIE, or CIE-WD mice were similarly unaffected. sIPSC frequency (5.4 ± 1.5, 5.7 ± 1.1, and 5.1 ± 1.9 Hz; *p *=* *0.9761 by one-way ANOVA; *n* = 3–7 cells from 3 mice/group; Fig. 9*C*, left), sIPSC amplitude (73.1 ± 5.0, 72.2 ±5.8, and 65.6 ± 0.5 pA; *p *=* *0.7758 by one-way ANOVA; *n* = 3–7 cells from 3 mice/group; Fig. 9*C*, middle), sIPSC rise (1.7 ± 0.7, 1.1 ± 0.1, and 1.0 ± 0.1; *p *=* *0.6595 by one-way ANOVA; *n* = 3–7 cells from 3 mice/group), and sIPSC decay (2.1 ± 0.1, 1.8 ± 0.1, and 2.4 ± 0.1 ms; *p *=* *0.0902 by one-way ANOVA; *n* = 3–7 cells from 3 mice/group; Fig. 9*C*, right) were all unchanged.

We also examined the tonic inhibitory conductance in CRF1^+^ and CRF1^−^ neurons after CIE and CIE-WD. Focal application of GBZ (100 μm) produced a significant reduction in holding current that was not significantly different among CRF1^+^ neurons from AIR, CIE, and CIE-WD mice (8.2 ± 1.4, 11.1 ± 2.1, and 8.7 ± 2.1 pA; *p *=* *0.5122 by one-way ANOVA; *n* = 5–7 cells from 3–4 mice/group; Fig. 9*D*,*E*). GBZ (100 μm) also produced a reduction in the amplitude of the holding current or rms noise that was not significantly different between CRF1^+^ neurons from AIR, CIE, and CIE-WD mice (10.3 ± 0.5–8.8 ± 0.6, 9.6 ± 0.7–8.3 ± 0.5, and 9.3 ± 0.7–8.5 ± 0.9 pA; *p *=* *0.4238 by one-way ANOVA; *n* = 5–7 cells from 3–4 mice/group]. Focal application of GBZ (100 μm) produced no reduction in holding current in CRF1^−^ neurons from AIR, CIE, or CIE-WD mice (0.7 ± 1.4, 2.2 ± 1.4, and 1.0 ± 3.6 pA; *p *=0.7642 by one-way ANOVA; *n* = 3–7 cells from 3 mice/group; Fig. 9*F*), no difference in the magnitude of reduction in the amplitude of the holding current or rms noise (10.0 ± 0.7–9.1 ± 0.6, 10.2 ± 0.9–8.8 ± 0.4, and 7.8 ± 0.1–6.6 ± 0.4 pA; *p *=* *0.6785 by one-way ANOVA; *n* = 3–7 cells from 3 mice/group) and no significant difference among the experimental groups. These data suggest that tonic inhibitory signaling in the LA is insensitive to chronic ethanol exposure and chronic ethanol exposure followed by withdrawal.

### *In vivo* microdialysis

**Figure 10.**
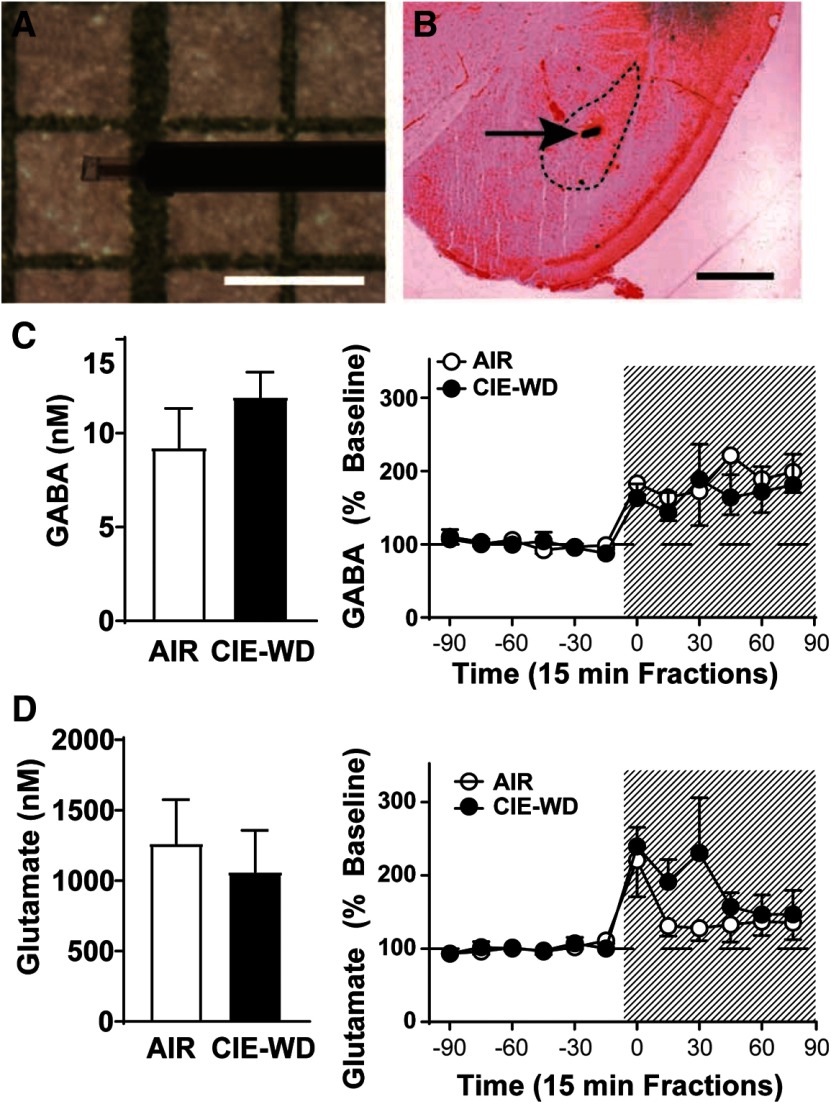
Effects of chronic ethanol vapor and withdrawal on exogenous GABA and glutamate concentration and sensitivity to acute ethanol in lateral amygdala/basolateral amygdala. ***A***, Representative microdialysis probe (0.5 mm). Scale bar, 1 mm. ***B***, Histologic verification of probe site. Dashed lines indicate LA/BLA. Scale bar, 1 mm. ***C***, Baseline dialysate concentrations of GABA (nm, left) and percent change in GABAergic transmission over time and following reverse dialysis of ehthanol (1 M, shaded area; right) in the LA/BLA of AIR and CIE-WD mice (*n* = 4–7). ***D***, Baseline dialysate concentrations of glutamate (nm, left) and percent change in glutamatergic transmission over time and following reverse dialysis of ehthanol (1 M, shaded area; right) in the LA/BLA of AIR and CIE-WD mice (*n* = 4–7).

To evaluate baseline transmitter levels following chronic ethanol exposure and withdrawal, we performed *in vivo* microdialysis in CRF1:GFP mice exposed to AIR (*n* = 4) or CIE-WD (*n* = 7). Mice were implanted with 0.5 mm microdialysis probes (Fig. 10*A*) aimed at the LA. However, as some penetrance into BLA is possible, results are described as LA/BLA (Fig. 10*B*). There were no significant differences detected between AIR and CIE-WD mice in basal GABA levels (9.2 ± 2.1 and 11.9 ± 1.3 nm; *p *=* *0.28 by unpaired *t* test; *n* = 4–7; Fig. 10*C*). Acute administration of ethanol (1 m) in the perfusate solution produced significant increases in LA/BLA GABA levels in both AIR and CIE-WD mice as assessed by two-way ANOVA of pre-ethanol and postethanol reverse dialysis (exposure condition × time) with a significant main effect of time (*F*_(11,99)_ = 5.585, *p *=* *0.0001), but no significant effect of exposure condition or interaction of time and exposure condition (Fig. 10*D*). There were also no significant differences detected between AIR and CIE-WD mice in basal glutamate levels (1264 ± 310.5 and 1061 ± 295.8 nm; *p *=* *0.67; *n* = 4–7; Fig. 10*E*). Acute administration of ethanol (1 m in the perfusate solution) produced significant increases in LA/BLA glutamate levels in both AIR and CIE-WD mice as assessed by two-way ANOVA of pre-ethanol and postethanol reverse dialysis (exposure condition × time) with a significant main effect of time (*F*_(11,99)_ = 4.747, *p *=* *0.0001), but no significant effect of exposure condition or interaction of time and exposure condition (Fig. 10*F*). These data suggest that baseline excitatory and inhibitory transmitter levels in the LA/BLA are not significantly altered by chronic ethanol exposure and withdrawal, and that the responsivity of these transmitter systems to ethanol also remains intact following chronic ethanol exposure and withdrawal.

## Discussion

The CRF1 system in the amygdala has been shown to play an important role in the development of ethanol dependence, but the CRF1^−^-containing neuronal population specifically within the LA has not been fully characterized. Here, we report that CRF1^+^ neurons in the LA are composed of multiple subgroups, including a small percentage of neurons expressing calcium binding proteins and a larger percentage of glutamatergic neurons. CRF1^+^ neurons exhibit distinct membrane properties, minor differences in baseline excitability, and possess an ongoing tonic GABA_A_ receptor conductance that CRF1^−^ neurons lack. Acute ethanol exposure increases the inhibition of CRF1^−^ neurons, but the inhibitory control of CRF1^+^ neurons is insensitive to acute ethanol. CRF1^+^ neurons displayed increased excitability following chronic ethanol; however, neither CRF1^+^ nor CRF1^−^ LA cells displayed alterations in phasic or tonic GABAergic synaptic transmission following chronic ethanol exposure or withdrawal, and basal changes in extracellular GABA or glutamate levels were not observed between exposure groups. Collectively, these results suggest that CRF1^−^ LA neurons are sensitive to acute ethanol but that changes in CRF1^+^ neuronal excitability following chronic ethanol are not due to neuroplastic changes in inhibitory control.

Both phasic and tonic GABAergic signaling regulate the activity and output of amygdala neurons. CRF1^+^ LA cells exhibit heightened basal phasic GABAergic signaling compared with CRF1^−^ cells and an ongoing tonic conductance that CRF1^−^ cells lack. Subunit stoichiometry regulates the ability of GABA_A_ receptors to mediate tonic inhibition, with the δ, α5, and ε subunits imparting sensitivity of GABA_A_ receptors to low levels of GABA that are thought to underlie tonic conductance (Stell and Mody, 2002; Stell et al., 2003; Glykys and Mody, 2007).The results of the immunohistochemical studies indicate that CRF1^+^ cells predominantly express the α1 subunit and exhibit little colocalization with the δ subunit, consistent with previous reports (Wiltgen et al., 2009). Consistent with this observation, the tonic conductance seen in this population was insensitive to application of the δ-preferring GABA_A_ receptor agonist THIP. The tonic GABA_A_ receptors in CRF1^+^ cells of the LA therefore do not contain δ subunits but may contain alternative subunit stoichiometry, such as α1β2γ2 or α5βγ2. In the CeA, the tonic conductance exhibited by CRF1^+^ neurons was enhanced by the application of the preferential α1 GABA_A_ agonist zolpidem, suggesting a role for α1-containing GABA_A_ receptors in tonic inhibition in that population. A similar mechanism may regulate tonic conductance in LA CRF1^+^ neurons. The δ subunit was sparsely expressed in unlabeled LA cells, as seen previously (Pirker et al., 2000), and a tonic conductance in CRF1^−^ neurons was stimulated by acute application of THIP. These findings suggest that CRF1^−^ cells express δ subunit-containing GABA_A_ receptors that are not active under basal conditions but may be stimulated by agonist activity or heightened concentrations of extracellular GABA.

Previous research has assessed the effects of ethanol on inhibitory signaling within the LA/BLA broadly, but the effects of ethanol on GABAergic signaling and within specific CRF1^+^ and CRF1^−^ populations have not been previously assessed. We observed that CRF1^+^ cells are relatively insensitive to changes in inhibitory control induced by acute ethanol; focal application failed to elicit a change in either phasic or tonic inhibitory signaling in this population. As CRF1^+^ neurons exhibited heightened phasic and tonic GABA_A_ signaling, these results may suggest a ceiling effect that precludes the possibility of GABA mimetics such as ethanol from further increasing sIPSC frequency or reducing holding current. In contrast, CRF1^−^ cells demonstrated an increased tonic conductance in the presence of ethanol coupled with a significant increase in GABA release onto these cells. These differences in sensitivity to acute ethanol may be related to GABA_A_ subunit expression differences between the two populations. δ-Containing GABA_A_ receptors have heightened sensitivity to ethanol (Wallner et al., 2003; Wei et al., 2004), and the δ-expressing CRF1^−^ neurons exhibited increases in tonic inhibitory control in response to ethanol that the δ-lacking CRF1^+^ cells failed to demonstrate. The insensitivity of CRF1^+^ cells to acute ethanol was also observed in the CeA (Herman et al., 2013), suggesting that this population may have similar GABA_A_ receptor compositions in multiple amygdala nuclei.

In contrast to the selective effects of acute ethanol, both phasic and tonic GABA_A_ signaling in LA CRF1^+^ and CRF1^−^ cells were not affected by chronic ethanol exposure or ethanol exposure and withdrawal. The microdialysis experiments showed that chronic ethanol and withdrawal did not produce adaptations in extracellular GABA or glutamate levels, which may explain the insensitivity of tonic conductance in CRF1^−^ neurons to ethanol-induced adaptations. Chronic ethanol exposure has been shown to increase basal GABA concentration in the CeA (Roberto et al., 2004a), elevating ambient GABA that is thought to drive cell type-specific changes in inhibitory control (Herman et al., 2016). Although the GABA_A_ receptor subunits associated with CRF1^+^ and CRF1^−^ neurons in the CeA and LA are similar, the lack of elevated ambient GABA in the LA likely precludes any chronic ethanol-induced plasticity in inhibitory signaling in either CRF1^+^ or CRF1^−^ LA neurons. Together, these findings may suggest that, unlike the CeA, inhibitory control of CRF1^+^ neurons in the LA is relatively preserved following chronic ethanol exposure.

Importantly, following chronic ethanol exposure CRF1^+^ neurons displayed reductions in the rheobase and threshold to fire, indicating increased excitability of CRF1^+^ neurons but not CRF1^−^ neurons. Thus, although inhibitory signaling in the CRF1^+^ population is relatively insensitive to the effects of acute ethanol, it is sensitive to chronic ethanol in multiple amygdala nuclei (the CeA and LA), making the CRF1^+^ population an important target for the actions of ethanol broadly within the amygdala. The results of the voltage-clamp experiments suggest that this enhanced excitability in the CRF1^+^ population is not driven by alterations in GABAergic signaling, which may indicate that these changes are instead regulated by ethanol-induced alterations in intrinsic excitability within the LA. Plasticity in glutamatergic signaling within the LA/BLA has been reported following chronic ethanol exposure (McCool et al., 2010), and the LA specifically exhibits alterations in molecular markers of glutamate signaling following chronic ethanol exposure in nonhuman primates (Alexander et al., 2018) and reinstatement of alcohol seeking in mice (Salling et al., 2017). Future work to characterize glutamatergic signaling in the CRF1^+^ and CRF1^−^ populations of the LA both under basal conditions and following chronic ethanol exposure would help to clarify the mechanisms underlying these ethanol-induced changes in excitability.

In the chronic vapor exposure experiments, we did not find evidence for increased baseline phasic GABAergic signaling in CRF1^+^ versus CRF1^−^ cells that was observed in our experiments with& naive mice. The baseline sIPSC frequency in CRF1^+^ neurons from AIR, CIE, and CIE-WD mice was lower than what was observed in CRF1^+^ neurons from naive mice and higher in CRF1^−^ neurons from AIR, CIE, and CIE-WD mice (Figs. 4*A*,*B*, 9*B*,*C*), collectively leading to a loss of significant differences between the two cell populations in the chronic ethanol exposure experiments. As our data indicate that the CRF1^+^ cell population is composed of a majority of glutamatergic principal neurons and a smaller subpopulation of interneurons, it is possible that differences in cell subpopulations sampled between the two experiments could account for these different baseline characteristics. However, we did observe tonic inhibition in the CRF1^+^ population and not the CRF1^−^ population in slices from both naive and vapor-exposed mice, which suggests that a similar population of cells was sampled in both sets of experiments. The loss of population differences in phasic but not tonic inhibition may be attributable to the stress of repeated injection, as the air-exposed mice were given daily pyrazole injections as a control for the treatment given to the CIE and CIE-WD groups. It is also possible that exposure to the air chamber, which as a novel environment may be a mild stressor, contributed to the differences between naive and AIR mice in these experiments. The relative sensitivity of phasic and tonic inhibitory control in CRF1^+^ cells to repeated mild stress is an interesting avenue for future studies to explore.

Together, these findings suggest that, unlike adaptations in inhibitory control exhibited by other amygdala nuclei (notably the CeA), GABAergic signaling within the LA is intact despite chronic ethanol exposure and/or withdrawal. This resistance to ethanol-induced plasticity in inhibitory control within the LA may play a significant role in the development of alcohol dependence and alcohol use disorders. Sensory information, including external drug cues and internal states such as craving and withdrawal, is relayed first to the LA from the cortex and thalamus; glutamatergic projections from the LA then synapse with CeA, BLA and lateral paracapsular neurons. Our findings suggest that, despite chronic ethanol exposure, inhibitory control of LA CRF1^+^ neurons (many of which are projection neurons) remains unchanged, allowing these cells to communicate with downstream amygdalar regions unimpeded. This suggests that neuroadaptations developing in the CeA (Herman et al., 2016) and BLA (Läck et al., 2007; Diaz et al., 2011) on chronic ethanol exposure result from local, intrinsic changes rather than from changes in extrinsic inputs from the LA. These findings may have relevance to amygdala circuitry in other contexts, such as fear learning, and may provide insights into other diseases involving amygdala dysfunction, including anxiety and depression. These findings also highlight the heterogeneous cell types within the LA and underscore the need for further cell type-specific characterization of amygdala physiology and pathology.

## Synthesis

Reviewing Editor: Alexxai Kravitz, Washington University in St. Louis Decisions are customarily a result of the Reviewing Editor and the peer reviewers coming together and discussing their recommendations until a consensus is reached. When revisions are invited, a fact-based synthesis statement explaining their decision and outlining what is needed to prepare a revision will be listed below. The following reviewer(s) agreed to reveal their identity: Julia Lemos.

The two reviewers raised several methodological issues with the paper and reporting that should be addressed before publication. It is expected that the resulting work would take no more than 2 months to complete, although if you require more time that is fine. We have synthesized the concerns into three main points, which should be addressed in a resubmission.

1. The biggest concern of both reviewers was the lack of information on the recording parameters and methods in multiple places throughout the manuscript. Specifically, comments 4, 5, 7, 8, 9, and minor comments i, ii, iii, iv, vi, x, and xi from Reviewer 1, and comments 1-10 under the “Experimental” heading from Reviewer 2 should be addressed in full.

2. The second concern related to the identity of the CRF1+ neurons, and whether it can be assumed that the CRF1-GFP mouse accurately captures CRF1 expressing neurons. Comments 2 and minor comment v from Reviewer 1, and comment 2 under the “Structure” heading from Reviewer 2 brought this up. Specifically, there was also some confusion around Figure 6, which appears to have been done in CRF1-GFP mice, and the green channel is presented for RNAscope, but there is no CRF1-GFP cell bodies in the images. Regardless of the images in this specific figure, the identity of the CRF1-GFP neurons was a major issue for the reviewers and should be addressed, possibly with a new experiment counting the overlap in CRF1-GFP and CRF mRNA if it is not possible to answer this with existing data.

3. Both reviewers also had suggestions for improving the framing and readability of the manuscript. These include comments Comments 1, and minor comments vii. Viii. Ix, xii and xiii of Reviewer 1, and comments 1 under the “Structure” heading, and 1 and 2 under the “Discussion” heading of Reviewer 2. Some of these are stylistic and are ultimately up to the authors to decide what they prefer, but we encourage you to consider these viewpoints of the reviewers.

--------------------------------------------

Original reviews

Review 1:

The authors examined the physiology and effects of ethanol in different neurons within the lateral amygdala. A genetically-encoded marker for the CRF receptor-1 was used to differentiate the different neuronal subtypes. Neuronal excitability as well as synaptic transmission were examined in the different subtypes, and ethanol effects on tonic and phasic GABAergic synaptic transmission was also assessed. Standard electrophysiological and pharmacological approaches, as well as in vivo microdialysis were used. The findings from the experiments described in the manuscript are clear and interesting for the most part. However, the rationale for the specific experiments is not clear, and a few additional experiments would help to support some of the conclusions. In addition, more information and possibly additional data should be included for the reported experiments.

Major Comments:

1) It is not clear why the authors chose to use CRF1 as the marker to separate different neuronal subtypes in LA, especially since effects of CRF itself were not examined and yet alcohol/CRF interactions were highlighted in the introduction. While this genetically-encoded marker appears to identify different cell types, other markers or additional markers might provide a more informative separation. The rationale for using this particular marker should be better developed in the introduction.

2) It would be helpful if the authors had an independent technique to assess CRF1 expression (e.g. an antibody to the receptor or RNAscope). As the authors probably know, genetically-encoded markers only reveal if the promoter has been activated. The expression of CRF1 mRNA in LA is equivocal in the Justice (2008) paper, but they only present low resolution images, so that experiment should be revisited with more sensitive approaches.

4) It is surprising that the authors didn’t quantify the number of action potentials or rheobase for the firing of the different neuronal subtypes. This information could be added in the discussion related to figure 1.

5) Was cell counting performed using stereological techniques? If not, this can lead to inadvertent double-counting of the same somata.

7) The data in Figure 3 panels C and F need additional explanation. It appears that somatic labeling is emphasized in panel C, while smaller puncta are shown in panel F. Is that the case? If so, what was the a rationale for focusing on these two different neuronal subcompartments for the different GABAA subunits? Also, quantification of colocalization in this figure (as in figure 5) would be helpful.

8) Why was there no measure of RMS noise in the ethanol experiments shown in figure 7?

9) Page 19, the description of the AIR and CIE data relative to naïve mice indicates an effect of the pyrazole injection, the chamber/air exposure or both. Do the authors know which factor produces the alteration? It could be relevant to effects of stress.

Minor Comments:

i. The authors need to indicate the temperature at which recordings were performed. This could certainly affect neuronal activity and tonic GABA levels. This is especially important for experiments involving Y-tube application of drugs, as that would change the local temperature unless both recording and application were at the same temp (i.e. solution applied through the Y-tube was preheated to match the bath temperature).

ii. The sequences of the probes form ACD Biotechne could be included in supplementary material.

iii. How was the 1 M ethanol concentration chosen for the reverse dialysis experiments? What tissue concentrations of ethanol are induced by this treatment?

iv. The number of sIPSCs used for event analysis is on the low side. The authors should provide cumulative probability histograms (at least for representative recordings) to indicate the distribution of these currents.

v. Regarding figure 5, did CB, CR and PV label different populations of neurons? If so, the total percentage of neurons expressing any of these proteins could be useful in corroborating the VGLUT1 labeling-based estimate of how many projection neurons express CRF1.

vi. More information should be provided about the target retrieval procedure.

vii. Page 23, last paragraph, the studies cited examined BLA not LA, so it is unclear if this discussion has much relevance to the present study.

viii. The comparisons to CeA do not add much to the discussion. These amygdala subregions are quite different in terms of neuronal phenotypes, and thus there is no rationale to compare neurons other than the fact that they express the same receptor.

ix. In general the discussion is too long, and could be shortened by eliminating the detailed description of phasic and tonic GABAergic transmission (bottom of page 21). Reducing the comparison of LA and CeA neurons, as mentioned above, will also be helpful. Other reductions could also be considered. For example, the discussion of the potential microcircuitry on page 22 is speculative without additional experiments.

x. Figure 1A, is the last column resting membrane potential? The neurons do not fire spontaneously, correct?

xi. Figure 7A, current records with a faster time scale should also be shown to help the reader see the sIPSCs and the effect of acute ethanol in the CRF1- neurons.

xii. Page 5, 2nd-to-last paragraph, please spell out Zeitgeber instead of just writing ZT. The full name of THIP should also be spelled out upon first use.

xiii. In general, the authors include too many significant digits in the reporting of physiological measures. For most of these measurements the precision is in the 0.1 to 0.01 range.

Reviewer 2:

The manuscript entitled “Corticotropin releasing factor receptor-1 neurons in the lateral amygdala display selective sensitivity to acute and chronic ethanol exposure” examines neurochemical and physiological differences between CRF1+ and CRF1- neurons in the lateral amygdala using the CRF1::GFP transgenic mouse. In addition, the study examines differential sensitivity of CRF1¬+ and CRF1- LA neurons to bath application of EtOH and in vivo exposure to chronic intermittent ethanol using vapor chambers. The study uses a multi-disciplinary approach to examine the nature and function of CRF1+ and CRF1- neurons in LA. This is a well done study of broad interest to the field. The study is generally well thought out, experiments are well executed and the statistics are well done. I have a few additional analyses and suggestions that would improve the manuscript. Once they are addressed, this manuscript with be suitable for publication.

EXPERIMENTAL:

1. Based on Figure 1b,c, there is some indication that CRF1+ cells show spike accommodation compared to CRF1-. Please indicate if this is the case across the population. Please report those results and statistics. If not, replace Figure 1b with something more representative.

2. The data in 1a indicate that CRF1+ neurons are considerably more excitable, with a trend toward higher Rm. What was the rheobase for each cell type? Please report these data and stats.

3. Was the KCl intracellular solution used for current clamp recordings. It is unusual to do measures of cell excitability with this type of intracellular solution. Usually a K-gluconate or K-MeSO4 based internal is used for these measurements. Please specify and justify if using KCl solution in the current clamp recordings.

4. Pg 13, Results: Why is the effect size reported as a negative value as opposed to absolute value?

5. Pg13, Results: RMP effect size should be reported in mVs not, MΩs.

6. Pg 13, Results: Just checking that there was not a significant difference in input resistance?

7. Specify if sIPSCs are TTX-sensitive.

8. 60 events is relatively low for fitting decay kinetics. In the future, consider using >200.

9. IPSCs should be fit with a double exponential in MiniAnalysis to capture fast and slow kinetics.

10. Report the basal holding currents of CRF1+ and CRF1- neurons in addition to the GZ-sensitive current.

STRUCTURE:

1. Thematically Figure 1d,e (and traces) seem to go more with Figure 2. Consider making Figure 1 - membrane properties, excitability and Figure 2 - phasic and tonic GABA transmission.

2. The study finds that CRF1+ cells are primarily glutamatergic neurons, yet where these data are placed within the structure of the paper, in effect, hides this main result. The authors should consider placing this at the beginning of “phenotype of CRF1+ LA cells”. The authors should also consider placing that section at the beginning of the paper since it gives context to the whole rest of the story.

DISCUSSION

1. Give a quick summary of the findings at the end of “Expression of GABA-A subunits” results section for readers that are not GABA-A subunit experts. I would also explicitly say that even though CRF1+ neurons have larger tonic current, that it is not mediated by delta-subunit containing GABA-A receptors - and then give alternative stoichiometry that may underlie the enhanced tonic GABA current observed in CRF1+ cells (i.e. α5βγ2).

2. In the discussion, consider that there may be a ceiling effect under these conditions, that may preclude CRF1+ cells from responding to GABAmimetics such as EtOH.

## Author Response

The two reviewers raised several methodological issues with the paper and reporting that should be addressed before publication. We expect that the resulting work would take no more than 2 months to complete, although if you require more time that is fine. We have synthesized the main concerns into three main points with both I and the reviewers agreed should be addressed in a resubmission.

1. The biggest concern of both reviewers was the lack of information on the recording parameters and methods in multiple places throughout the manuscript. Specifically, comments 4, 5, 7, 8, 9, and minor comments i, ii, iii, iv, vi, x, and xi from Reviewer 1, and comments 1-10 under the “Experimental” heading from Reviewer 2 should be addressed in full.

We have clarified methods and added additional analyses as requested by the reviewers throughout the Results section. See below for a detailed response to each comment.

2. The second concern related to the identity of the CRF1+ neurons, and whether it can be assumed that the CRF1-GFP mouse accurately captures CRF1 expressing neurons. Comments 2 and minor comment v from Reviewer 1, and comment 2 under the “Structure” heading from Reviewer 2 brought this up. There was also some confusion around Figure 6, which appears to have been done in CRF1-GFP mice, and the green channel is presented for RNAscope, but there is no CRF1-GFP cell bodies in the images. Regardless of this specific figure, the identity of the CRF1-GFP neurons was a major issue for the reviewers and should be addressed, possibly requiring a new tissue experiment counting the overlap of GFP and CRF1 mRNA in the CRF1-GFP mouse, if it is not possible to answer this with existing data.

We agreed with both reviewers that validation of the GFP signal in the LA of the CRF1:GFP mouse is an important consideration. We have added a new tissue experiment as requested to address these concerns, using RNAscope to confirm the fidelity and penetrance of the GFP label in the CRF1+ neuronal population in the LA. This information is included in the revised manuscript.

3. Both reviewers also had suggestions for improving the framing and readability of the manuscript. These include Comments 1, and minor comments vii. Viii. Ix, xii and xiii of Reviewer 1, and comments 1 under the “Structure” heading, and 1 and 2 under the “Discussion” heading of Reviewer 2. Some of these are stylistic and are ultimately up to the authors to decide what they prefer, but we encourage you to consider these viewpoints of the reviewers.

We agreed with these comments and have incorporated the suggestions made by the reviewers into our revisions. Significant changes have been made to the composition and order of figures as well as the overall structure of the manuscript, with particular attention to streamlining the Discussion section. We feel the readability of the manuscript is significantly improved thanks to these changes.

Reviewer 1:

The authors examined the physiology and effects of ethanol in different neurons within the lateral amygdala. A genetically-encoded marker for the CRF receptor-1 was used to differentiate the different neuronal subtypes. Neuronal excitability as well as synaptic transmission were examined in the different subtypes, and ethanol effects on tonic and phasic GABAergic synaptic transmission was also assessed. Standard electrophysiological and pharmacological approaches, as well as in vivo microdialysis were used. The findings from the experiments described in the manuscript are clear and interesting for the most part. However, the rationale for the specific experiments is not clear, and a few additional experiments would help to support some of the conclusions. In addition, more information and possibly additional data should be included for the reported experiments.

Major Comments:

1) It is not clear why the authors chose to use CRF1 as the marker to separate different neuronal subtypes in LA, especially since effects of CRF itself were not examined and yet alcohol/CRF interactions were highlighted in the introduction. While this genetically-encoded marker appears to identify different cell types, other markers or additional markers might provide a more informative separation. The rationale for using this particular marker should be better developed in the introduction.

The introduction has been edited to more clearly state the rationale for the use of the CRF1 neuronal population in these experiments (pages 3-4).

2) It would be helpful if the authors had an independent technique to assess CRF1 expression (e.g. an antibody to the receptor or RNAscope). As the authors probably know, genetically-encoded markers only reveal if the promoter has been activated. The expression of CRF1 mRNA in LA is equivocal in the Justice (2008) paper, but they only present low resolution images, so that experiment should be revisited with more sensitive approaches.

We agree that the ability to reliably differentiate CRF1+ and CRF1- neurons is an important consideration. We have included a new experiment to quantify CRF1 mRNA in the LA of the reporter mouse line using RNAscope and determine the extent of colocalization between CRF1 and GFP mRNA. We report 74% of Crhr1+ neurons co-express Gfp and 84% of Gfp+ neurons co-express Crhr1 (Figure 1C), indicating significant penetrance and fidelity, respectively. These findings are depicted in revised Figure 1 and are detailed in the Results section (pages 12-13).

4) It is surprising that the authors didn't quantify the number of action potentials or rheobase for the firing of the different neuronal subtypes. This information could be added in the discussion related to figure 1.

We have included new analyses of excitability for each cell type in the CRF1+ and CRF1- populations from the naïve mice (revised Figure 3). We did not observe significant differences in rheobase between these populations, but CRF1+ neurons did exhibit an increased threshold to fire. We also observed potential ethanol-induced changes in rheobase and threshold to fire in the CRF1+ but not CRF1- neurons in the vapor exposure experiments (revised Figure 8). These results are now stated in the Results (pages 15 and 19-20) and considered in the Discussion (pages 22-23 and 25-26).

5) Was cell counting performed using stereological techniques? If not, this can lead to inadvertent double-counting of the same somata.

RNAscope is a semi-quantitative technique identifying nuclei localization, not absolute quantification of soma. Imaging and analysis for RNAscope is often performed using widefield microscopy of 5-20um thick sections without stereological techniques, including the protocol from the company (Wang et al., 2012). We used confocal microscopy of z-stacks with a thickness of 5um (Wang et al., 2012) which decreases the likelihood of overlapping nuclei. Our method for identification of expressing cells is semi-quantitative and only signal co-localized within DAPI/nuclei staining was analyzed to increase confidence in detection.

7) The data in Figure 3 panels C and F need additional explanation. It appears that somatic labeling is emphasized in panel C, while smaller puncta are shown in panel F. Is that the case? If so, what was the rationale for focusing on these two different neuronal subcompartments for the different GABAA subunits? Also, quantification of colocalization in this figure (as in figure 5) would be helpful.

In all cases, GFP labeling was typically somatic, and a neuron was only considered as CRF1+ if GFP expression was observed throughout the soma. GABAA receptor subunits did display selective expression on the membrane or in intercellular/extrasynaptic compartments that was observed as smaller, more discontinuous puncta (revised Figure 5C and 5F), making it difficult to reliably dissociate between cells. Thus, we feel the GABAA receptor subunit expression information is more useful as a qualitative measure of spatial distribution and expression in CRF1+ neurons.

8) Why was there no measure of RMS noise in the ethanol experiments shown in figure 7?

RMS noise was measured in these experiments and found to be insensitive to ethanol exposure. This information is now included in the revised text (page 18).

9) Page 19, the description of the AIR and CIE data relative to naïve mice indicates an effect of the pyrazole injection, the chamber/air exposure or both. Do the authors know which factor produces the alteration? It could be relevant to effects of stress.

The effects of the chamber as well as the pyrazole as a mediator of the differences between naïve and AIR mice is an important consideration. The design of the present experiments does not allow us to dissociate the effects of the air chamber and pyrazole injection, so either or both variables could be responsible for the differences between the naïve and AIR mice observed here. We have expanded the treatment of these variables in the discussion section (pages 26-27) to include chamber exposure as another potential contributor to these differences.

Minor Comments:

i. The authors need to indicate the temperature at which recordings were performed. This could certainly affect neuronal activity and tonic GABA levels. This is especially important for experiments involving Y-tube application of drugs, as that would change the local temperature unless both recording and application were at the same temp (i.e. solution applied through the Y-tube was preheated to match the bath temperature).

All recordings for these experiments were performed at room temperature, with both the bath and solutions applied via the y-tube in unheated containers. This is now indicated in the Methods section (page 5).

ii. The sequences of the probes form ACD Biotechne could be included in supplementary material.

RNAscope's probe design varies from traditional ISH such that there is no single sequence for each probe. RNAscope employs a probe design strategy in which two independent probes (double Z probes) have to hybridize the target sequence in tandem in order for signal amplification to occur. Each of these probes is 18-25 bases long and complementary to the target RNA, and for each RNA about 20 double Z probes are designed to hybridize to the target RNA covering a roughly 1kb long target region depending on the design. This makes the probes incredibly specific. The manufacture's website does not provide the probe sequence for ~40 Z probes per RNA, but rather the large target region covering about 1kb of the RNA depending on the probe/RNA. We now report the target region for each probe in the Methods section (page 8).

iii. How was the 1 M ethanol concentration chosen for the reverse dialysis experiments? What tissue concentrations of ethanol are induced by this treatment?

This dose was chosen in order to be consistent with prior experiments utilizing reverse dialysis of ethanol in rodents (Roberto et al., 2004a, PMID 14973247; Roberto et al., 2004b, PMID 15537886 ). In this work, a 1M dose of ethanol was found to induce maximal changes in extracellular GABA and glutamate levels. We have updated the Methods section (page 10) to include this rationale for the ethanol dose used.

iv. The number of sIPSCs used for event analysis is on the low side. The authors should provide cumulative probability histograms (at least for representative recordings) to indicate the distribution of these currents.

The total number of events analyzed was dependent on the frequency of events and the time period analyzed. 65 events is the minimum number of events for a cell to be included in the analyses in these experiments to allow for the inclusion of cell types that have lower baseline sIPSCs. However, events for these experiments ranged from 300-500 per cell during baseline recordings. Cumulative probability histograms for the acute ethanol experiments are included in the revised manuscript (revised Figure 7).

v. Regarding figure 5, did CB, CR and PV label different populations of neurons? If so, the total percentage of neurons expressing any of these proteins could be useful in corroborating the VGLUT1 labeling-based estimate of how many projection neurons express CRF1.

We did not examine colocalization of the different CBPs in the present experiments. In rat, there is significant overlap of the LA CB+ and PV+ populations (PMID: 10992249, PMID: 11166127), so a subtractive approach to these targets is likely not appropriate. As other papers in the LA have demonstrated variability in the relationship between CBPs and GABA neurons (PMID: 11516833, PMID: 10992249), we included the VGLUT1 experiments to definitively determine the percentage of CRF1 neurons that were glutamatergic. The CBP experiments are informative about the diverse cell types within the CRF1 population but do not conclusively identify a CRF1 neuron as GABAergic. Language in the relevant section of the results (page 13) and discussion (pages 23-24) sections has been tempered to better reflect the relative contributions of the CBP and VGLUT1 experiments.

vi. More information should be provided about the target retrieval procedure.

We have updated the methods section (pages 7-8) with a detailed description of the target retrieval procedure.

vii. Page 23, last paragraph, the studies cited examined BLA not LA, so it is unclear if this discussion has much relevance to the present study.

We agree with the reviewer and have removed this paragraph.

viii. The comparisons to CeA do not add much to the discussion. These amygdala subregions are quite different in terms of neuronal phenotypes, and thus there is no rationale to compare neurons other than the fact that they express the same receptor.

We have removed many of the details about the CeA as suggested throughout the discussion section. We have left a few references to prior work in CeA as this work specifically characterized tonic conductance and ethanol sensitivity in the CRF1+ neuronal population. We feel the remaining references are useful as a basis for comparison in describing CRF1+ neurons generally, and informs the discussion with respect to the lack of chronic ethanol effects in the LA.

ix. In general the discussion is too long, and could be shortened by eliminating the detailed description of phasic and tonic GABAergic transmission (bottom of page 21). Reducing the comparison of LA and CeA neurons, as mentioned above, will also be helpful. Other reductions could also be considered. For example, the discussion of the potential microcircuitry on page 22 is speculative without additional experiments.

We agree with the reviewer and have substantially revised the discussion into the most relevant points. We have removed the details about phasic and tonic transmission, most references to the CeA, and the speculation about microcircuitry, as suggested.

x. Figure 1A, is the last column resting membrane potential? The neurons do not fire spontaneously, correct?

The last column is resting membrane potential. We did not observe spontaneous firing in these experiments, but we did not record spontaneous firing activity in whole-cell current clamp configuration. All current injection experiments were conducted at -70mV in order to eliminate spontaneous firing.

xi. Figure 7A, current records with a faster time scale should also be shown to help the reader see the sIPSCs and the effect of acute ethanol in the CRF1- neurons.

We have included new traces with a faster time scale to allow better visualization of the sIPSCs in these experiments (revised Figure 7).

xii. Page 5, 2nd-to-last paragraph, please spell out Zeitgeber instead of just writing ZT. The full name of THIP should also be spelled out upon first use.

Zeitgeber is now written in full as requested. THIP's full name is stated in text on first use (page 4).

xiii. In general, the authors include too many significant digits in the reporting of physiological measures. For most of these measurements the precision is in the 0.1 to 0.01 range.

Significant digits throughout the manuscript have been adjusted to show values in the 0.1-0.01 range.

Reviewer 2:

The manuscript entitled “Corticotropin releasing factor receptor-1 neurons in the lateral amygdala display selective sensitivity to acute and chronic ethanol exposure” examines neurochemical and physiological differences between CRF1+ and CRF1- neurons in the lateral amygdala using the CRF1::GFP transgenic mouse. In addition, the study examines differential sensitivity of CRF1+ and CRF1- LA neurons to bath application of EtOH and in vivo exposure to chronic intermittent ethanol using vapor chambers. The study uses a multi-disciplinary approach to examine the nature and function of CRF1+ and CRF1- neurons in LA. This is a well done study of broad interest to the field. The study is generally well thought out, experiments are well executed and the statistics are well done. I have a few additional analyses and suggestions that would improve the manuscript. Once they are addressed, this manuscript with be suitable for publication.

EXPERIMENTAL:

1. Based on Figure 1b,c, there is some indication that CRF1+ cells show spike accommodation compared to CRF1-. Please indicate if this is the case across the population. Please report those results and statistics. If not, replace Figure 1b with something more representative.

We have included new analyses of spike accommodation in the CRF1+ and CRF1- populations from naïve mice (revised Figure 3). Consistent with the exemplar traces, 90% of CRF1+ neurons exhibited spike accommodation versus only 52% of CRF1- neurons. These population differences were conserved in neurons from mice used for the vapor exposure experiment (revised Figure 8). These findings are now detailed in the Results (pages 14 and 18-20) and Discussion (pages 23 and 26) sections.

2. The data in 1a indicate that CRF1+ neurons are considerably more excitable, with a trend toward higher Rm. What was the rheobase for each cell type? Please report these data and stats.

We have included new analyses of rheobase for each cell type in the CRF1+ and CRF1- populations from the naïve mice (revised Figure 3). We did not observe significant differences in rheobase between these populations, but CRF1+ neurons did exhibit an increased threshold to fire. We also observed potential ethanol-induced changes in rheobase and threshold to fire in the CRF1+ but not CRF1- neurons in the vapor exposure experiments (revised Figure 8). These results are now included in the revised manuscript as above.

3. Was the KCl intracellular solution used for current clamp recordings. It is unusual to do measures of cell excitability with this type of intracellular solution. Usually a K-gluconate or K-MeSO4 based internal is used for these measurements. Please specify and justify if using KCl solution in the current clamp recordings.

KCl intercellular solution was used for current clamp recordings that were carried out in conjunction with voltage clamp recordings of sIPSCs and tonic conductance. The use of an intracellular solution containing high chloride is preferable for studies examining sIPSCs and tonic conductance and is necessary for comparison with previous work (see Herman et al. 2016, PMID 23426657.) Although the use of KCl intracellular solution does prevent the examination of changes in excitability within experimental recordings due to the inverted chloride reversal potential, it does not significantly impact baseline membrane or spiking properties.

4. Pg 13, Results: Why is the effect size reported as a negative value as opposed to absolute value?

All effect sizes are now reported as absolute values.

5. Pg13, Results: RMP effect size should be reported in mVs not, MΩs.

This error has been corrected (page 14).

6. Pg 13, Results: Just checking that there was not a significant difference in input resistance?

There is indeed a significant difference in resistance between CRF1+ and CRF1- LA neurons. We have updated revised Figure 3 and the Results section (page 14) to include this relevant finding.

7. Specify if sIPSCs are TTX-sensitive.

sIPSCs were not recorded in the presence of TTX to preserve intact network activity and facilitate comparisons with microdialysis studies.

8. 60 events is relatively low for fitting decay kinetics. In the future, consider using >200.

The total number of events analyzed was dependent on the frequency of events and the time period analyzed. 65 events is the minimum number of events for a cell to be included in the analyses in these experiments to avoid the exclusion of cell types that have lower baseline sIPSCs. However, the events for these experiments ranged from 300-500 sIPSCs per cell during baseline recordings.

9. IPSCs should be fit with a double exponential in MiniAnalysis to capture fast and slow kinetics.

Due to the inherent variability in exponential fit of sIPSC kinetics across all cell populations and the lack of significant changes in sIPSC kinetics in any of our experimental measures, we prefer to communicate the averaged kinetics data.

10. Report the basal holding currents of CRF1+ and CRF1- neurons in addition to the GZ-sensitive current.

These data are now reported in the Results section (page 15).

STRUCTURE:

1. Thematically Figure 1d,e (and traces) seem to go more with Figure 2. Consider making Figure 1 - membrane properties, excitability and Figure 2 - phasic and tonic GABA transmission.

We agree with the reviewer and have significantly restructured both the figures themselves and the order in which data appear. Membrane properties and excitability are now depicted in revised Figure 3, with phasic and tonic GABA transmission in revised Figure 4.

2. The study finds that CRF1+ cells are primarily glutamatergic neurons, yet where these data are placed within the structure of the paper, in effect, hides this main result. The authors should consider placing this at the beginning of “phenotype of CRF1+ LA cells”. The authors should also consider placing that section at the beginning of the paper since it gives context to the whole rest of the story.

We agree with the reviewer and have re-ordered the figures, the results and the discussion sections to put the genetic phenotype of CRF1+ LA neurons first (revised Figures 1 and 2), followed by the electrophysiological properties of these cells.

DISCUSSION

1. Give a quick summary of the findings at the end of “Expression of GABA-A subunits” results section for readers that are not GABA-A subunit experts. I would also explicitly say that even though CRF1+ neurons have larger tonic current, that it is not mediated by delta-subunit containing GABA-A receptors - and then give alternative stoichiometry that may underlie the enhanced tonic GABA current observed in CRF1+ cells (i.e. α5βγ2).

We agree with the reviewer that these are important points to include. The summary statement has been added to the results section as requested (page 17) and speculation about alternative stoichiometry has been added to the discussion (pages 23-24).

2. In the discussion, consider that there may be a ceiling effect under these conditions, that may preclude CRF1+ cells from responding to GABA mimetics such as EtOH.

This point has been added to the discussion section as requested (pages 24-25).
